# Oxide Semiconductor for Advanced Memory Architectures: Atomic Layer Deposition, Key Requirement and Challenges

**DOI:** 10.1007/s40820-025-02013-7

**Published:** 2026-01-05

**Authors:** Chi-Hoon Lee, Seong-Hwan Ryu, Taewon Hwang, Sang-Hyun Kim, Yoon-Seo Kim, Jin-Seong Park

**Affiliations:** 1 Division of Materials Science and Engineering, Hanyang University, 222 Wangsimni-ro, Seongdong-gu, Seoul, 04763 Republic of Korea; 2 Department of Display Science and Engineering, Hanyang University, 222 Wangsimni-ro, Seongdong-gu, Seoul, 04763 Republic of Korea; 3 Present Address: Imec, Kapeldreef 75, B-3001 Louvain, Belgium

**Keywords:** Oxide semiconductor (OS), Atomic layer deposition (ALD), Memory applications

## Abstract

Oxide semiconductors (OSs), introduced by the Hosono group in the early 2000s, have evolved from display backplane materials to promising candidates for advanced memory and logic devices. The exceptionally low leakage current of OSs and compatibility with three-dimensional (3D) architectures have recently sparked renewed interest in their use in semiconductor applications. This review begins by exploring the unique material properties of OSs, which fundamentally originate from their distinct electronic band structure. Subsequently, we focus on atomic layer deposition (ALD), a core technique for growing excellent OS films, covering both basic and advanced processes compatible with 3D scaling. The basic surface reaction mechanisms—adsorption and reaction—and their roles in film growth are introduced. Furthermore, material design strategies, such as cation selection, crystallinity control, anion doping, and heterostructure engineering, are discussed. We also highlight challenges in memory applications, including contact resistance, hydrogen instability, and lack of p-type materials, and discuss the feasibility of ALD-grown OSs as potential solutions. Lastly, we provide an outlook on the role of ALD-grown OSs in memory technologies. This review bridges material fundamentals and device-level requirements, offering a comprehensive perspective on the potential of ALD-driven OSs for next-generation semiconductor memory devices.

## Highlights

- This review outlines the emergence of oxide semiconductors as promising channel materials for high-density, low-power next-generation memory applications.
- Adsorption and reaction mechanisms of atomic layer deposition have enabled the design of high-performance oxide semiconductors for next-generation memory applications.
- This review discusses key challenges toward successfully integrating oxide semiconductors into next-generation memory devices.

## Graphical abstract

**Figure:**
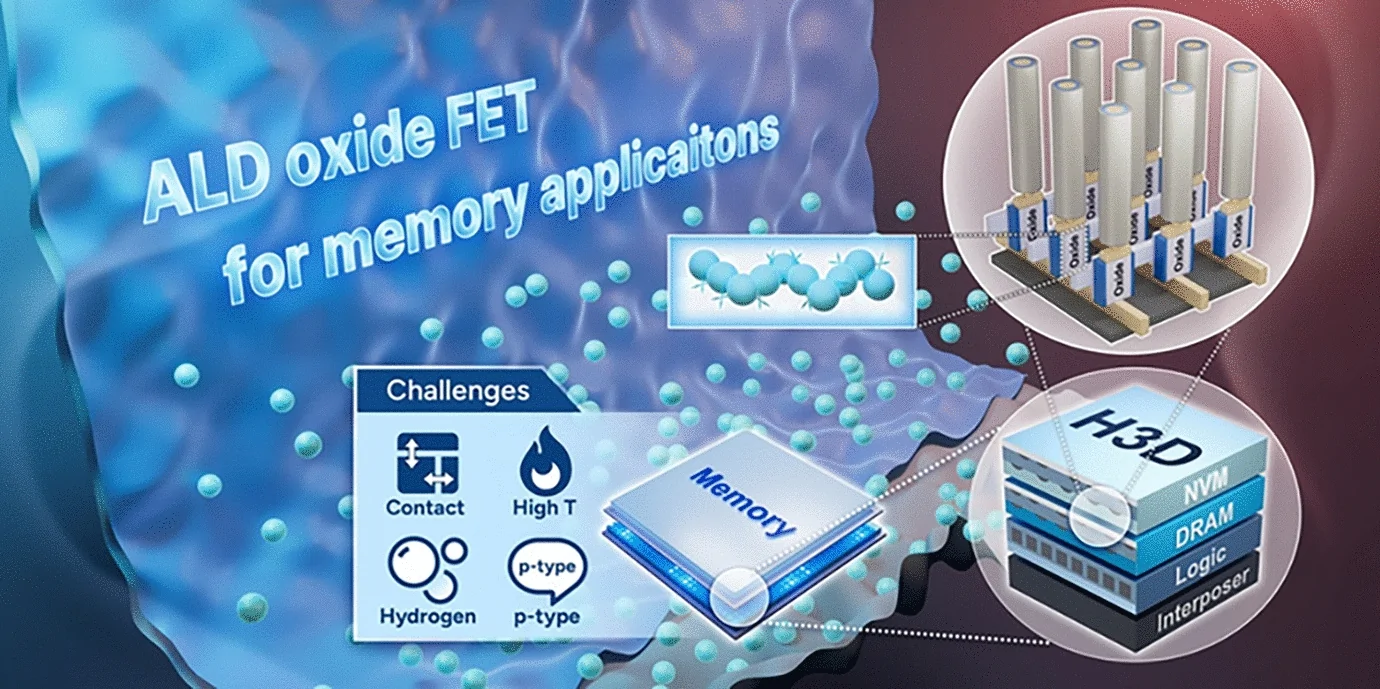

## Introduction

### Beyond Displays: OS Channels for Semiconductor Applications

Advances in modern memory and logic technologies have been driven by continuous scaling down of silicon-based devices. However, this progress is now facing physical limitations of device miniaturization and material limitations related to power consumption [1–4]. Consequently, the need for next-generation channel materials has become increasingly critical. Oxide semiconductors (OSs), well-known in the display industry for their excellent electrical properties and process compatibility, are now attracting growing interest in memory and logic applications [5–7]. Early research focused on their application in back-end-of-line (BEOL) logic technologies, given their capability for deposition on three-dimensional (3D) structures and low-temperature (below 400 °C) processing [8–10]. Recently, OSs have emerged as promising candidates for next-generation memory technologies, driven by demands for reduced cell size, increased transistor density, and vertical channel architectures to enhance integration density [11–13]. The low power consumption of these frameworks renders them especially attractive in dynamic random-access memory (DRAM) applications, where high leakage currents necessitate continuous dynamic refresh operations, leading to significant power consumption [12, 14]. In response to these industrial demands, extensive research is underway for the practical implementation of OS-based devices.

### OS Channel Roadmap: From Invention to Mass Production in Displays

**Fig. 1:**
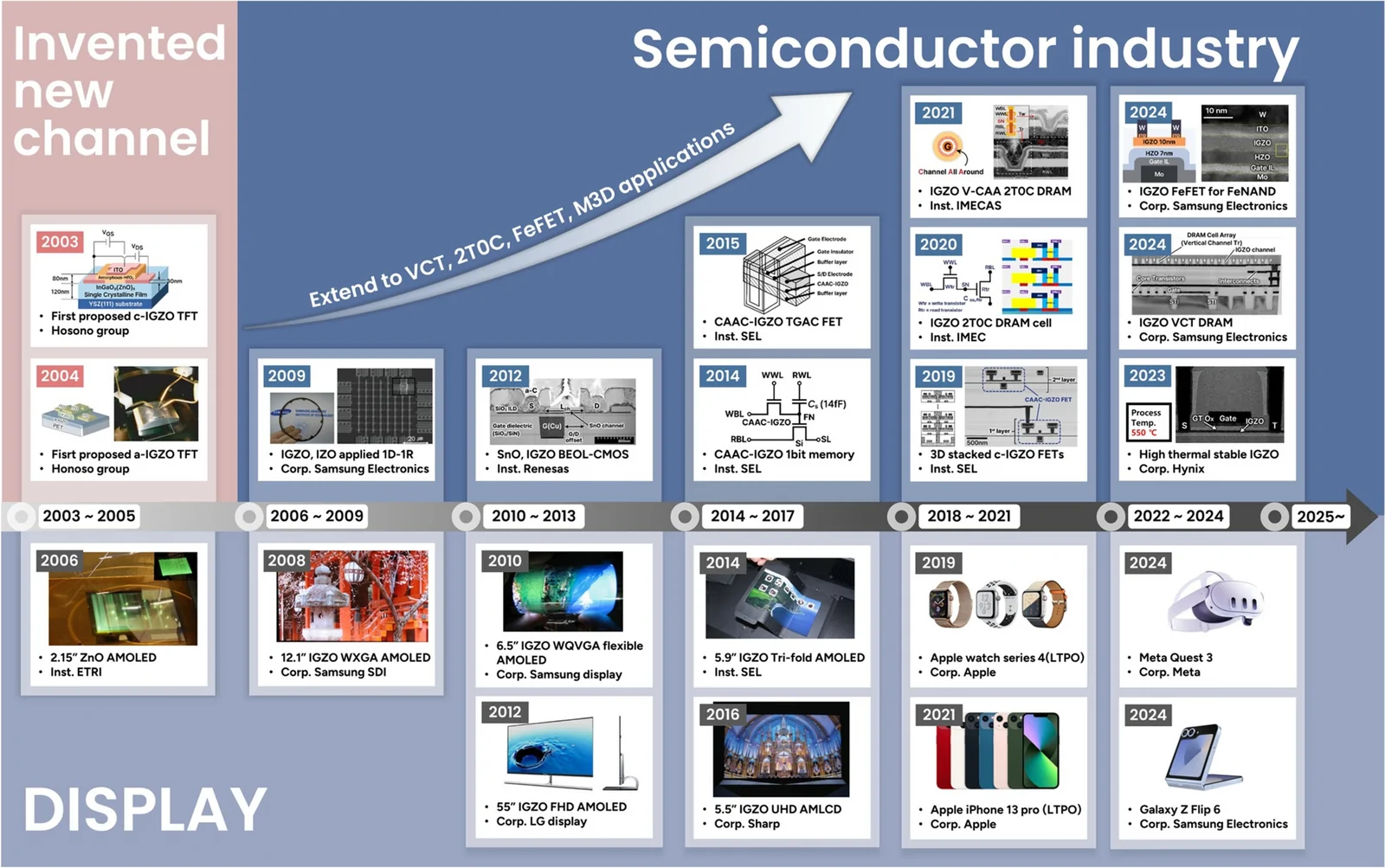
Chronological development and technological expansion of oxide semiconductor thin-film transistors (OS-TFTs) from display applications to high-performance memory and logic integration. Starting with the first proposal of IGZO-based TFTs, OS-TFTs were rapidly adopted in display technologies. Subsequently, the application scope has expanded to include advanced logic and memory devices. The images of Apple Watch Series 4 and Apple iPhone 13 Pro were provided by Apple, Inc. The images of Samsung Galaxy Z Flip 6 were provided by Samsung Electronics. Reproduced with permission [15–35]. Copyright 2003, Science. Copyright 2004, Nature. Copyright 2006, 2008, 2009, 2010, 2012, 2014, 2016, 2019, 2024, John Wiley & Sons. Copyright 2012, 2014, 2015, 2019, 2020, 2021, 2023, 2024, IEEE. Copyright 2023, Kim et al.

The idea of OSs as next-generation channel materials was first proposed by the Hosono group in 2003 through the demonstration of crystalline InGaZnO (IGZO; light red region in Fig. 1). Specifically, single-crystalline IGZO was synthesized via pulsed laser deposition (PLD) followed by annealing at 1400 °C. When used as the active layer in a thin-film transistor (TFT), the material exhibited a high field-effect mobility of approximately 80 cm^2^ V^−1^ s^−1^ [15]. In 2004, the same group demonstrated the potential of OSs for next-generation displays by fabricating amorphous IGZO (a-IGZO) TFTs on polyethylene terephthalate substrates at room temperature (RT) using PLD and validating their transfer characteristics [16].

Since Hosono’s introduction of a-IGZO, OSs have attracted growing interest in next-generation displays due to their low off-state current, high mobility, and low-temperature process compatibility (Fig. 1, light blue region). In 2006, the Electronics and Telecommunications Research Institute, Korea, demonstrated the first active matrix organic light emitting diode (AMOLED) panel using ALD-grown ZnO TFTs [17]. Subsequent milestones include Samsung SDI’s 12.1-in AMOLED with sputtered a-IGZO (2008) [18], and Samsung Display’s flexible AMOLED (2010) showing robust performance under 10,000 bending cycles [19]. In 2012, LG Display scaled IGZO to Gen. 8 glass for 55-in OLED TVs [20], while SEL introduced a foldable AMOLED using c-axis aligned crystalline indium-gallium-zinc oxide (CAAC-IGZO) in 2014 [21]. Sharp’s 1000 ppi IGZO liquid crystal display (LCD) (2016) enabled ultrahigh-resolution displays [22]. Commercial adoption followed, with Apple integrating IGZO into the Apple Watch (2019) and iPhone 13 Pro (2021) [23, 24]. Most recently, IGZO has been adopted in Meta Quest 3 and Samsung Galaxy Z Flip 6 (2024), highlighting its scalability and commercial viability [25].

### OS Channel Roadmap for Semiconductor Industrial Applications

As shown in the dark blue region in Fig. 1, research into the application of OSs in the memory and logic industries began expanding in 2009 with Samsung Electronics Inc.’s report on an 8 × 8 one-diode-one-resistor array based on oxide transistors. In this work, IGZO was used to prepare the selector transistor, and indium-zinc oxide (IZO) was applied as the diode. ALD-processed NiO demonstrated superior properties compared with sputtered NiO for the resistive element [26]. In 2012, Renesas Corp. demonstrated, for the first time, SnO TFTs exhibiting an *I*_on_/*I*_off_ ratio over 10^4^ and a drain voltage capability exceeding 40 V, highlighting the feasibility of SnO and IGZO-based BEOL-complementary metal-oxide semiconductor (CMOS) I/O integration within conventional silicon-based large-scale integration processes [27]. In 2014, SEL Institute realized a novel 50 nm-scale field-effect transistor (FET) employing CAAC-IGZO, reporting excellent device characteristics with a drain-induced barrier lowering (DIBL) of 67 mV V^−1^ and a subthreshold swing (SS) of 92 mV dec^−1^. Circuit simulations further demonstrated that memory devices based on this FET architecture could achieve write speeds below 5 ns and retention times exceeding 1000 s [28]. In 2015, SEL reported a scalable, low-cost trench-gate-self-aligned CAAC-IGZO FET fabricated with only three masks at the 20 nm node, achieving a DIBL of 0.12 V V^−1^ and an SS of 97 mV dec^−1^ [29]. In 2019, SEL introduced a CAAC-IGZO FET with a gate length of 72 nm integrated into a 3D monolithic stack, fabricated via a trench-gate self-aligned flow at ≤ 400 °C with a top-/back-gate effective oxide thickness (EOT) of ~ 6/ ~ 35 nm and a back-gate for *V*_th_ control (− ∂*V*_th_/∂*V*_bg_ ≈ 0.13 V/V), with the thermal budget tuned to preserve lower-/upper-device performance. Simulation results indicated the potential for non-volatile OS random-access memory operation featuring write speeds under 10 ns and endurance beyond 10^12^ cycles [30]. In 2020, IMEC Institute reported a world-first capacitor-less two-transistor (2T0C) architecture based on IGZO FETs, built in a 300-mm back-end-of-line (BEOL)-compatible flow using a bottom oxygen-channel stack (SiO_2_ under IGZO with an Al_2_O_3_ top gate), O_2_-anneal-driven defect passivation, and contact/layout engineering (ALD-TiN to suppress O-scavenging and minimized extension length) to scale the gate-dielectric EOT and boost C_ox_, achieving retention times exceeding 400 s without the need for a capacitor [31]. In 2021, IMECAS experimentally demonstrated the world’s first vertical channel-all-around (CAA) IGZO FET structure within a 2T0C DRAM cell, built in a BEOL-compatible PEALD flow at ~ 250 °C using O_2_ plasma to deposit IGZO/Al_2_O_3_/IZO in situ, with Mo/SiO_2_/Mo vertical MIM S/D defining the critical dimension (CD); *V*_th_ and SS were optimized by adjusting the InO_x_:GaO_x_:ZnO_x_ cycle ratio and by lowering the plasma power (~ 38 W), with retention times exceeding 300 s [32]. In 2023, Hynix Corp. confirmed that crystalline IGZO maintained structural stability without agglomeration during a hydrogen-containing high-temperature process at 550 °C—conditions relevant to DRAM fabrication—whereas amorphous IGZO underwent degradation [33]. Most recently, in 2024, Samsung Electronics Inc. proposed a single-gate IGZO-based vertical channel transistor (VCT) structure, DRAM-oriented to suppress passing-gate interference below 10 nm, relocate BL/storage contacts for a 4F^2^ cell, favor ALD-IGZO over physical vapor deposition (PVD) for conformal channels with steeper SS and lower *I*_off_ at 85 °C, and bias composition toward Ga-rich/low-In to stabilize *V*_th_, overcoming limits of conventional Si-based DRAM and improving scalability [34]. Furthermore, the company demonstrated an oxygen-deficient IGZO-based ferroelectric FET (FeFET), which exhibited a large memory window of 17.8 V and a fast pulse response of approximately 1 µs [35]. Overall, the widespread adoption of OSs in display technologies has not only demonstrated their technological significance but also accelerated their exploration for use in next-generation memory applications.

### Growing Attention for OS Channel in Semiconductor Applications

**Fig. 2:**
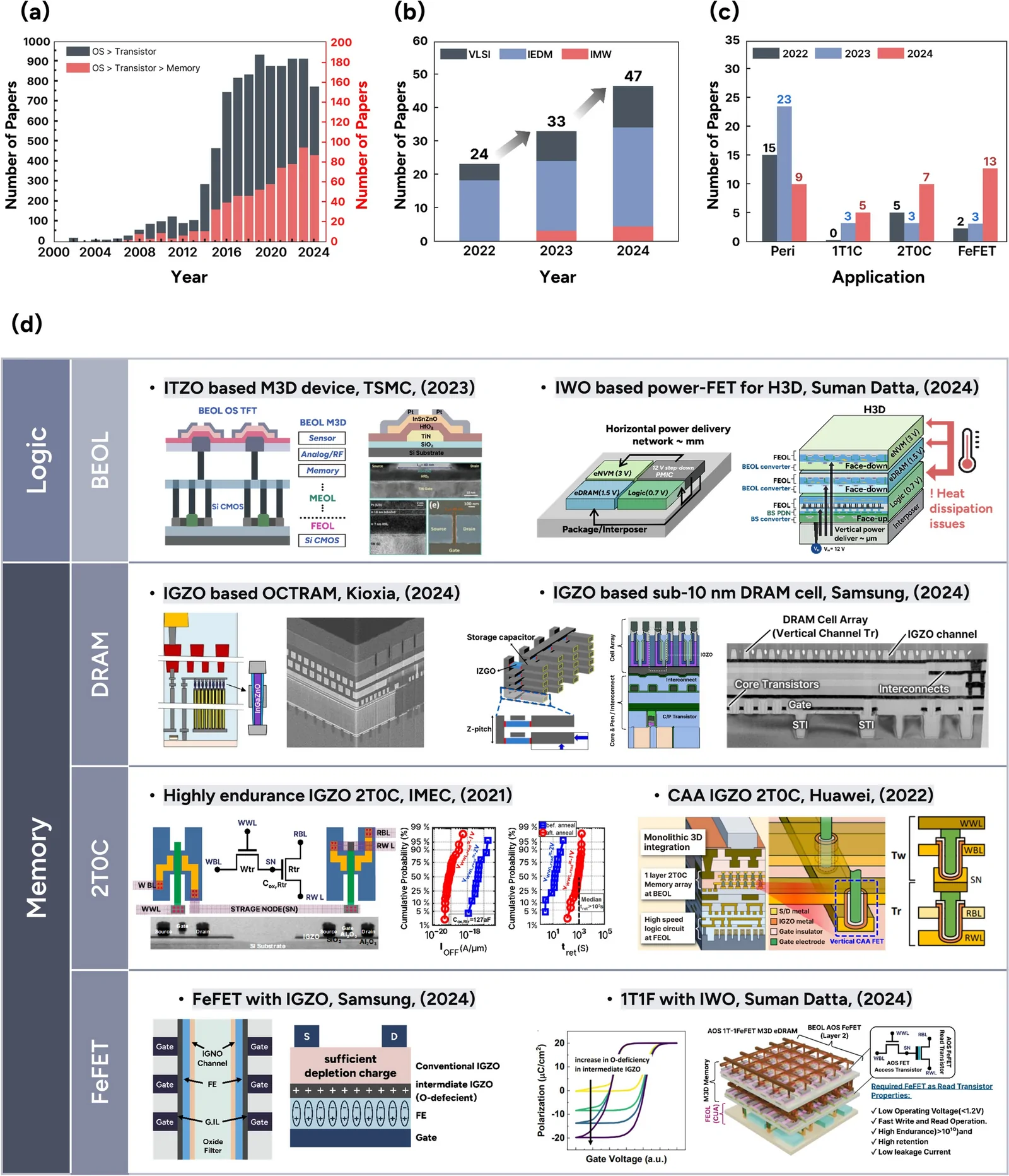
Recent research trends and application landscape of oxide–semiconductor-based devices for logic and memory applications. **a** Annual increase in the number of publications and cumulative citations on oxide semiconductors for memory applications, indicating growing research interest. **b** Yearly distribution of relevant papers across major conferences (IEDM, VLSI, IMW) from 2022 to 2024. **c** Number of published works by application category—back-end-of-line (BEOL), 1T1C, 2T0C, and FeFET—showing increased focus on memory. **d** Representative device demonstrations categorized by application. Reproduced with permission [12, 34, 35, 53, 65, 81, 89, 92]. Copyright 2021, 2022, 2023, 2024, IEEE

The rising volume of research publications highlights the growing interest in OSs for memory applications. Figure 2a illustrates the chronological increase in the number of publications related to OS devices. The marked acceleration in memory-focused research since 2020 highlights the increasing relevance of OSs in the field. Figure 2b summarizes the number of papers on OSs presented annually at major international conferences, including the International Memory Workshop (IMW), International Electron Devices Meeting (IEDM), and Symposia on VLSI Technology and Circuits (VLSI). A clear upward trend can be observed from 2022 to 2024, with the total number of papers rising from 24 in 2022 to 47 in 2024, reflecting the expanding attention garnered by this field within the memory and logic industries. Figure 2c categorizes the annual publication count from 2022 to 2024 by application type—BEOL, one-transistor one-capacitor (1T1C), 2T0C, and ferroelectric field-effect transistor (FeFET)—within the memory and logic sectors. While BEOL-related publications increased from 15 in 2022 to 23 in 2023, a decline to 9 was observed in 2024. In contrast, the number of publications related to 1T1C, 2T0C, and FeFET increased to 5, 7, and 13, respectively, in 2024. In other words, although early research on OSs in logic circuits primarily focused on BEOL integration, a recent shift toward their use as core cell transistors in memory applications has been observed. The device benchmarks for each application can be found in Tables 1 and 2 [36–86].

**Table 1:** Benchmark of OS FETs for BEOL applications (VLSI/IEDM/IMW, 2022–2024)

OS channel	Channel thickness (nm)	Process method	Cell architecture	Thermal budget (°C)	V_th_ (V)	μ_FET_ (cm^2^ V^−1^ s^−1^)	S.S (mV dec^−1^)	PBS ΔV_th_ (V)	Refs
In_2_O_3_	3.1	ALD	GAA	250	− 2.6	76–86	100	− 0.7	[36]
	1.9–2.5	ALD	Planar	300	0.1	55	150–185	–	[37]
	1.5–1.8	ALD	Planar	235	0	–	163	–	[38]
	1.5, 2.2	ALD	Planar	450	− 1.1	107	100–150	–	[39]
	2.5	ALD	Planar	225	− 2.5	–	100	− 0.07	[40]
	1.3–2.0	ALD	Planar	225	0	> 100	–	–	[41]
	1.6	ALD	Planar	400	> 0	72	70	0.03	[42]
	1.2–2.0	ALD	Buried-gate	290	–	62	100	–	[43]
	2.5	ALD	Planar	350	0	175	75	–	[44]
In_2_O_3_	3.5	ALD	Planar	250	0	45	65	0.02	[45]
	2	ALD	Planar	300	0.1	60	69	0.03	[46]
InGaO	3	ALD	Planar	225	0.4	29	85	0.03	[47]
	7	ALD	Nanosheet	600	0	30	100	0.1	[48]
	–	ALD	Planar	250	0	–	75	0.08	[49]
	3	ALD	Planar	300	1.2	14	80	–	[50]
	–	ALD	Planar	450	0	20–100	68–75	–	[51]
	2.9	ALD	Planar	400	0.5	26	95	− 0.07	[52]
InSnO	–	ALD	Planar	400	–	–	–	–	[53]
	8	ALD	GAA	400	0	22	68–75	− 0.09	[54]
InGaZnO	3.0–4.0	ALD	Planar	250	0	30	75	0.12	[55]
	2	ALD	Planar	200	–	–	–	0.5	[56]
	10	ALD	Planar	250	0	13	126	–	[57, 58]
	1.5	ALD	Planar	250	0	–	68	0.05	[59]
	15	ALD	VCT	300	0	–	< 100	–	[60]
	6	ALD	V-GAA	400	0.1	–	75–80	− 0.02	[61]
	5.6	ALD	Planar	250	0	–	60	− 0.1	[62]

**Table 2:** Benchmark of OS FETs for 1T1C, 2T0C, and FeFET (VLSI/IEDM/IMW, 2022–2024)

Device category	OS channel	Process method	Cell architecture	I_on_ (μA μm^−1^)	S.S (mV dec^−1^)	Memory Windo^1^)	Retention time (s)	Endurance (cycle)	Multi-level conductance (bit)	Refs
1T1C	IGZO	–	VCT	> 150.0	–	–	3600	–	–	[63]
1T1C	In_2_O_3_	PVD	VCT	112.2	84	–	> 100	–	–	[64]
2T0C	IGZO	ALD	CAA	32.8	92	–	–	–	–	[65]
2T0C	IGZO	ALD	CAA	4.1	109	10^6^	190	10^12^	–	[66]
2T0C	IGZO	ALD	CFET	–	135	–	200	500	2	[67]
2T0C	IGZO	ALD	CAA	> 3.0	190	> 10^6^	100	10^11^	–	[68]
2T0C	IGZO	ALD	Planar	> 10 − 6	68	–	10^4^	–	4	[69]
2T0C	IGZO	ALD	Planar	–	65	–	> 5000	–	–	[70]
2T0C	IGZO	PVD	Planar	1500	–	–	> 300	–	3	[71]
2T0C	IGZO	PVD	Planar	270	75	10^5^	1000	–	4	[72]
2T0C	IGZO	ALD	CAA	0.2	135	25	20	–	–	[73]
2T0C	IGZO	PVD	Planar	∼4.0	–	10^6^	180	–	–	[74]
2T0C	IGZO	PVD	Planar	24	–	5	> 10^4^	–	3	[75]
2T0C	IGZO	PVD	Planar	10.6	76	10^5^	> 300	–	3	[76]
FeFET	In_2_O_3_	ALD	Planar	–	–	2.5	–	> 10^9^	–	[77]
FeFET	In_2_O_3_	ALD	VCT	17	65	1.8	> 1000	> 10^9^	–	[78]
FeFET	In_2_O_3_	ALD	Planar	100	68–105	–	> 1000	10^7^	–	[79]
FeFET	IGO	ALD	GAA	–	90	2.5	> 104	–	2	[80]
FeFET	IWO	ALD	Planar	1	97	1	> 104	> 10^12^	–	[81]
FeFET	IGZO	ALD	Planar	0.2	–	> 1	> 105	> 10^10^	–	[82]
FeFET	IGZO	PVD	Planar	–	–	0.7	–	10^7^	–	[83]
FeFET	IGZO	PVD	Planar	∼2	–	3.3	–	10^10^	–	[84]
FeFET	IGZO	PVD	Planar	–	–	10	> 1000	10^9^	3	[85]
FeFET	IGZO	PVD	Planar	∼1	–	17.8	10^3^	> 10^4^	–	[35]
FeFET	IGZO	PVD	Planar	1.2	62	2.1	> 10^8^	> 10^7^	–	[86]

^1^For 2T0C, memory window is defined as ${I}_{read}/{I}_{off}$. For FeFET, memory window is defined as${V}_{th}^{program}-{V}_{th}^{erase}$

In the evolution of OS-channel-based memory technologies, three cell primitives—1T1C, 2T0C, and FeFET—are drawing significant attention. 1T1C stores charge on a discrete capacitor written/sensed through a gated access path. The defined cell capacitor yields a predictable bit-line signal (Δ*V*≈*Q*/*C*_BL_) and controllable retention, while physical separation of the storage node from the channel mitigates disturb and variability, enabling a stable, reproducible memory framework based on destructive read followed by immediate restore [87, 88]. 2T0C is a capacitor-less dynamic cell that separates write/store and read-sense with two transistors, where the parasitic capacitance of read transistor serves as the storage node; removing the capacitor eases BEOL integration and shrinks pitch, with long retention, sensing margin, and variability as key challenges [31, 89]. FeFET embeds a ferroelectric material in the OS gate stack to realize non-volatile, polarization-driven *V*_th_ shifts, enabling fast, low-energy operation and analog programmability for in-memory computing [90, 91]. Across these, OS channels are receiving growing attention in next-generation semiconductor development, with sustained academic–industrial efforts.

Recent studies on OS channels for memory and logic applications can be categorized into four types—BEOL, 1T1C, 2T0C, and FeFET—as shown in Fig. 2d. In the context of logic-oriented BEOL integration, from a high-density scaling perspective, in 2023, TSMC Ltd. reported the implementation of a 1.8-nm ultrathin indium-tin-zinc-oxide film—deposited via ALD—as the channel material in a FET with a 40-nm channel length. This device demonstrated a low DIBL of 22 mV V^−1^ along with a respectable field-effect mobility of 48 cm^2^ V^−1^ s^−1^ [92]. From a BEOL operational validation and guidance perspective, in 2024, the Suman Datta group investigated the bias-temperature instability (BTI) characteristics of high-voltage W-doped In_2_O_3_ (IWO)-based power FETs designed for heterogeneous 3D (H3D) systems. Using machine learning models, the researchers predicted circuit reliability and proposed thermal optimization strategies, thereby establishing a technological foundation for energy-efficient circuit design in H3D system architectures [53]. Collectively, these findings underscore that BEOL deployment of oxide semiconductors hinges on aligning ALD-enabled device characteristics with thermally constrained, reliability-aware design practices in stacked architectures.

In the memory industry, OS channels have been actively investigated in the domains of 1T1C, 2T0C, and FeFET, with several recent studies reporting significant advancements. In the 1T1C category, considerable attention has been directed toward the use of the OS channel in VCTs. From an array-level demonstration perspective, in 2024, Kioxia demonstrated the world’s first high-density 4F^2^ DRAM (OCTRAM) featuring a 275 Mbit array, integrating a gate-all-around (GAA) IGZO VCT above a high-aspect-ratio capacitor structure, using a capacitor-first stack to decouple capacitor/VCT interactions and a ~ 26 nm vertical hole with ALD-IGZO. Device performance was optimized by selecting contact materials to avoid interfacial oxide, thinning the gate oxide and spacer to boost the fringing field, and applying O_2_ anneal with channel-composition tuning to stabilize *V*_th_ through BEOL [12]. From a framework/roadmap and process-guidance perspective, in the same year, Samsung Electronics proposed two oxide-based device architectures—an IGZO VCT and a vertically stacked cell array transistor (VS-CAT)—to address the scaling limits of sub-10-nm DRAM technology. Their comparative analysis of PVD and ALD processes for IGZO VCTs highlighted the significance of process optimization, while the use of IGZO in the VS-CAT architecture enabled epitaxy-free stacking without a seed layer, offering advantages such as *z*-pitch reduction and process simplification [34]. Collectively, Kioxia’s array-level validation and Samsung’s framework-level guidance jointly establish both feasibility and a credible technology path for OS channel VCTs as a leading 1T1C DRAM option.

For the 2T0C architecture, research has focused on scaling down devices while improving electrical performance and reliability. From a planar IGZO-2T0C, architecture/integration-validation perspective, in 2021, IMEC Institute demonstrated the 2T0C DRAM operating at a 14 nm channel length using ALD-grown IGZO. Through the adoption of a gate-last integration scheme with a buried oxygen tunnel and careful optimization of the gate dielectric and IGZO channel thickness, the device achieved retention times exceeding 10^3^ s and endurance beyond 10^11^ cycles [89]. From a vertical CAA IGZO-FET, device-scaling/thermal-reliability perspective, in 2022, Huawei Technologies Co. fabricated the first vertical CAA IGZO FET with a sub-50-nm critical dimension using ALD. PEALD was employed to form conformal IGZO/HfO_x_/IZO (≈3/8/8 nm) and defining the ~ 55 nm channel length with a SiO_x_ spacer to strengthen electrostatics; the high-k HfO_x_ boosted C_ox_, while the stack preserved performance after 300 °C/30 min N_2_ anneal and up to 120 °C positive bias temperature stress (PBTS). This device demonstrated high-speed operation (*I*_on_ = 32.8 µA µm^−1^), low SS (92 mV dec^−1^), thermal reliability, and compatibility with 2T0C 4F^2^ cell architectures—positioning it as a key enabling technology for next-generation ultra-dense, low-latency 3D DRAM [65]. Collectively, these works establish a coherent advancement path for 2T0C, in which planar demonstrations provide array-level validation and integration guidance, and the vertical CAA device platform confirms scalable electrostatics and thermal reliability—jointly de-risking BEOL-compatible processing and paving the way toward ultra-dense 4F^2^ DRAM.

In the FeFET domain, recent efforts have emphasized reducing operating voltage, accelerating switching speed, and enhancing endurance. From a stack- and channel-engineering perspective aimed at maximizing the memory window and expediting the roadmap toward vertically integrated, high-density NVM, in 2024, Samsung Electronics Inc. achieved a record-high memory window of 17.8 V in an IGZO-based FeFET by strategically introducing an oxygen-deficient layer within the channel and engineering the gate interlayer. The device exhibited fast switching (1 µs), low-voltage operation, and the potential for multi-level signal storage, making it a promising candidate for future high-density non-volatile memory (NVM) [35]. From a BEOL-compatibility and embedded-systems viability perspective—prioritizing ultra-low-voltage operation and exceptional endurance to enable refresh-free 1 T–1FeFET, in 2023, the Suman Datta group developed an amorphous In_2_O_3_-based 1 T–1FeFET device that operated below 0.9 V, with a switching time of 20 ns, endurance over 10^12^ cycles. The potential for refresh-free embedded memory offers a new standard for NVMs in next-generation embedded DRAM and artificial intelligence (AI) accelerators [81]. Collectively, they strengthen the technological foundation linking materials/process innovation to system-level deployability across 3D NVM and energy-efficient embedded memory.

Particularly in AI system development, next-generation NVMs—FeFET, resistive random-access memory (RRAM), and phase change memory (PCM)—are pivotal as enablers of non-volatile on-chip weight storage, substantially reducing data-movement energy. Their multi-level conductance and fast, low-voltage switching enable compute-in/near-memory architectures for analog-like MACs and in situ learning.

In addition to FeFET, OS-channel FETs have been considered for use as selectors in resistive random-access memory (RRAM) or phase change memory (PCM), primarily because they combine low leakage with usable drive at logic-level biases and compatibility with BEOL fabrication. Recent BEOL stacks pair an ITO-engineered IGZO selector (*I*_on_ ≈ 196.5 µA µm^−1^, *I*_off_ ≈ 1 pA µm^−1^ at *V*_ds_ = 1 V) with a ~ 3.6-nm MoS_2_ switching layer to realize < 1 V, < 100 µA 1T1R operation and vertically stacked 2T0C1R hybrids [93]. Furthermore, sub-10-nm-L_ch_ ZnO selectors fabricated at ≤ 300 °C deliver record *I*_on_ ≈ 561 µA µm^−1^ with sub-pA/µm leakage and have been monolithically integrated with Al_2_O_3_-based RRAM into functional 1T1R arrays, underscoring OS-selector suitability for dense, energy-efficient crossbars [94].

Across BEOL, 1T1C, 2T0C, and FeFET platforms, high-aspect-ratio vertical channels have become a central focus of investigation. Work on structural design emphasizes precise control of channel geometry, carefully engineered spacer schemes, and conformal, void-free gate fill [95–100]. Efforts in channel-material engineering target composition tailoring, defect suppression, and improved interface passivation [97, 101–105]. Studies on source/drain contacts examine the role of contact interlayers and address resistance asymmetry [106–108]. Collectively, these advances accelerated the maturation of vertical-channel devices for next-generation semiconductor industry.

In summary, OSs continue to attract interest for BEOL integration in logic circuits owing to their low-temperature processability, while their role as core cell transistors in memory devices—particularly in 1T1C, 2T0C, and FeFET architectures—has emerged as a growing area of advanced research.

## Material Fundamentals of OS for Semiconductor Applications

Current semiconductor manufacturing faces interlinked bottlenecks—BEOL thermal budgets, overlay errors, inter-layer-via (ILV) pitch limits and interconnect RC overheads, selector/access-device off-state leakage, and scaled contact resistivity—that constrain integration density, and energy efficiency [109–116]. The low-temperature processability of OS channels supports M3D integration, lowering cumulative thermal budget and reducing overlay burden by in situ process. Monolithic proximity shortens interconnects and curtails parasitic capacitance, easing ILV pitch pressure and RC delay. In parallel, characteristically low leakage suppresses off-state conduction in selector/access devices, while native n-type behavior promotes electron accumulation at metal contacts without heavy doping or silicide control, reducing specific contact resistivity, in contrast to Si. Taken together, these intrinsic material characteristics indicate that OS channels can directly address several of these pain points.

### Intrinsic Characteristics of OSs for Semiconductor Applications

**Fig. 3:**
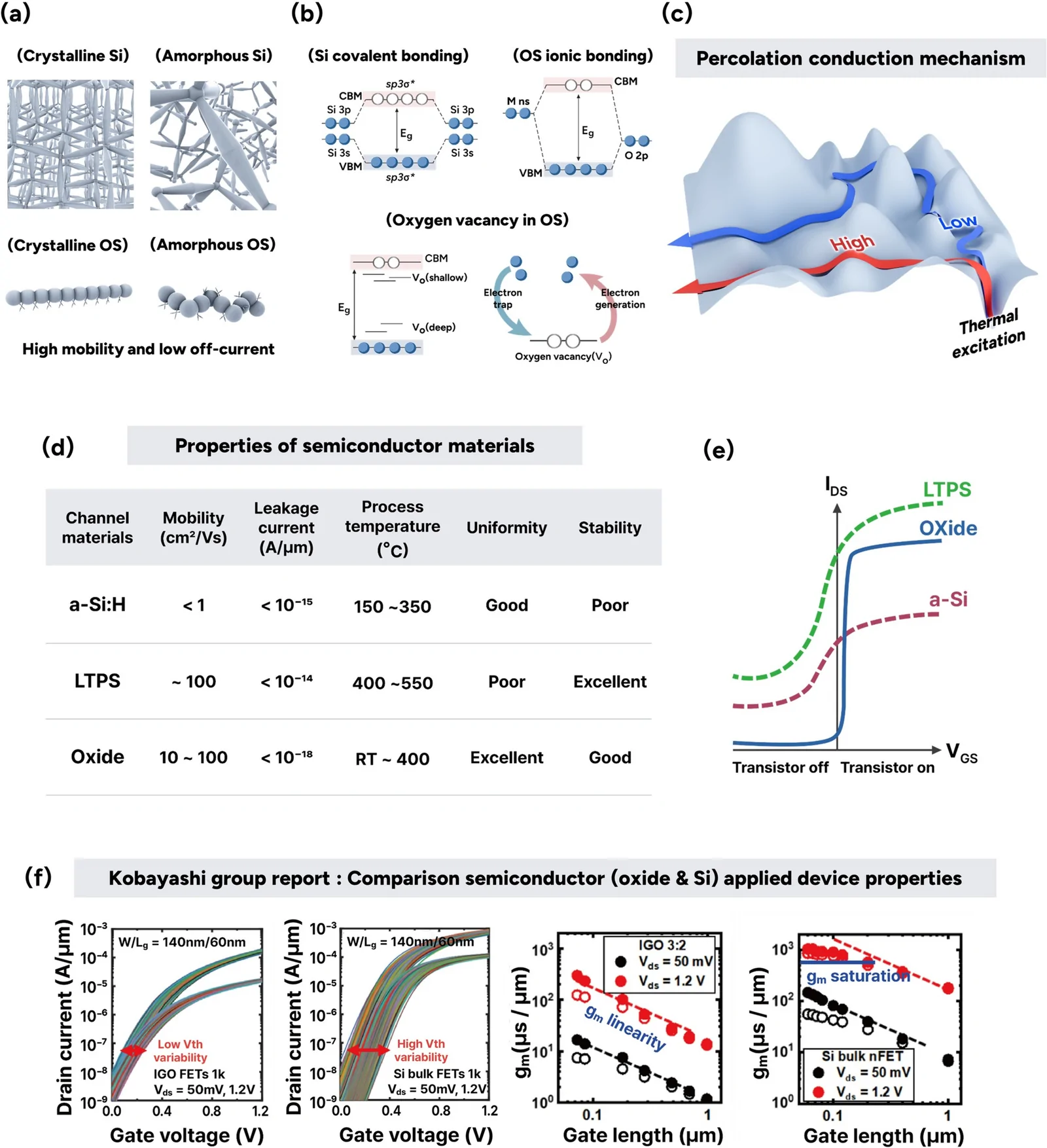
Comparison of structural, electronic, and device-level characteristics of oxide semiconductors (OSs) and silicon-based semiconductors. **a** Schematics of atomic configurations in crystalline and amorphous states for silicon and OSs. **b** Differences in energy band diagrams of Si and OS, including the role of oxygen vacancies. **c** Percolation conduction mechanism in OSs. **d** Comparison of key properties and **e** transfer curves for a-Si:H, LTPS, and OSs. **f** Kobayashi group benchmarking of oxide (IGO) and Si FETs. Reproduced with permission [123]. Copyright 2024, IEEE

The increasing interest in OSs for next-generation memory stems from their distinct electronic band structure, which enables low leakage current, 3D compatibility, and excellent electrical performance, as shown in Fig. 3. As shown in Fig. 3a, OSs maintain high electron mobility in the amorphous phase due to the isotropic and delocalized nature of metal ns orbitals at the conduction band minimum (CBM), enabling efficient charge percolation unlike the directional sp3 bonding in silicon. The CBM is dominated by metal ns orbitals with large radial extent, yielding a small transport effective mass (m* ≈ 0.2–0.35 m_0_) and suppressing band-edge localization despite topological disorder. As shown in Fig. 3b, the ionic bonding in OSs induces a wide bandgap between the O 2*p* valence band and metal ns CBM, enabling high transparency and low off-state leakage. Despite the large bandgap, oxygen vacancies act as key native defects that donate free electrons, thereby modulating the Fermi level and enabling n-type conductivity [8, 117–119]. These V_O_-related donor states are typically shallow (~ 0.1–0.3 eV below the CBM), so even modest thermal or electrostatic modulation can populate the conduction band. As shown in Fig. 3c, OSs exhibit carrier-dependent transport governed by percolation conduction. On this basis, OSs often show a temperature- and density-driven transition from variable-range hopping near the percolation threshold to band-like transport at higher carrier concentrations. In amorphous structures, the disorder creates local potential fluctuations, where free carriers—primarily from oxygen vacancies—facilitate charge transport. These vacancies introduce shallow donor levels that raise the Fermi level toward the CBM, increasing accessible conduction pathways. As the Fermi level nears the CBM, carrier delocalization enhances mobility, especially at higher carrier densities or temperatures, emphasizing the key role of defect chemistry in OS electrical performance [8, 16, 120, 121].

### Key Properties of Representative Channel Materials: A-Si:H, LTPS, and OS

Figure 3d compares key properties of representative channel materials, including a-Si:H, LTPS, and OS. a-Si:H enables low-temperature processing (150–350 °C) and good uniformity but suffers from low mobility (< 1 cm^2^ V^−1^ s^−1^) and poor stability. LTPS offers high mobility (~ 100 cm^2^ V^−1^ s^−1^), suitable for high-current applications, but its high crystallization temperature (400–550 °C) limits compatibility, and grain boundaries reduce uniformity across large areas. As shown in Fig. 3e, the high off-current of Si-based semiconductors limits their suitability for low-power memory. In contrast, OSs combine moderate-to-high mobility (10–100 cm^2^ V^−1^ s^−1^), ultralow leakage (~ 10^–18^ A µm^−1^), and low-temperature processability (RT–400 °C), making them ideal for display and memory applications [7, 16, 122]. Their steep on–off transitions and low off-currents enhance data retention and reduce refresh power, supporting their use as memory cell transistors.

### Benchmarking Report of OS Channel versus Si FETs

Recently, the Kobayashi group made a compelling case for the industrial viability of OS FETs in memory applications by benchmarking them against conventional Si bulk n-channel FETs. Figure 3f illustrates the strategic role of nanosheet OS FETs in enabling monolithic 3D integration, particularly for high-density and energy-efficient memory applications. The figure conceptually depicts the vertical stacking of logic and memory units, where oxide-based access transistors form the foundation of the memory layer directly integrated atop a logic wafer using BEOL-compatible processes. The unique advantages of OSs—high electron mobility, ultralow leakage current, and low-temperature processability—address the stringent requirements of 3D integration that traditional Si-based devices often fail to meet. In particular, recent studies on ALD-grown InGaO FETs have indicated that nanosheet devices with sub-100 nm gate lengths exhibit not only reliable operation at scaled dimensions but also unsaturated carrier velocity even at high fields, unlike conventional Si bulk FETs that exhibit early velocity saturation. This indicates strong potential for high-speed switching in densely packed vertical arrays. Moreover, statistical analyses of over 1000 fabricated nanosheet InGaO FETs reveal tight threshold voltage (*V*_th_) distributions (~ 20 mV), minimal DIBL (~ 18.7 mV V^−1^), and reduced variability in *I*_on_ (~ 4.8%), all of which are comparable or superior to those of foundry-grade Si bulk transistors. These findings underscore the feasibility of OSs in meeting the variability and reliability requirements for advanced memory-periphery or selector transistors in 1T1C/1T1R arrays [123].

## ALD for OSs: Trends, Fundamentally Engineering, and Advanced Developments

### Rising Focus on ALD for OSs in Semiconductor Applications

**Fig. 4:**
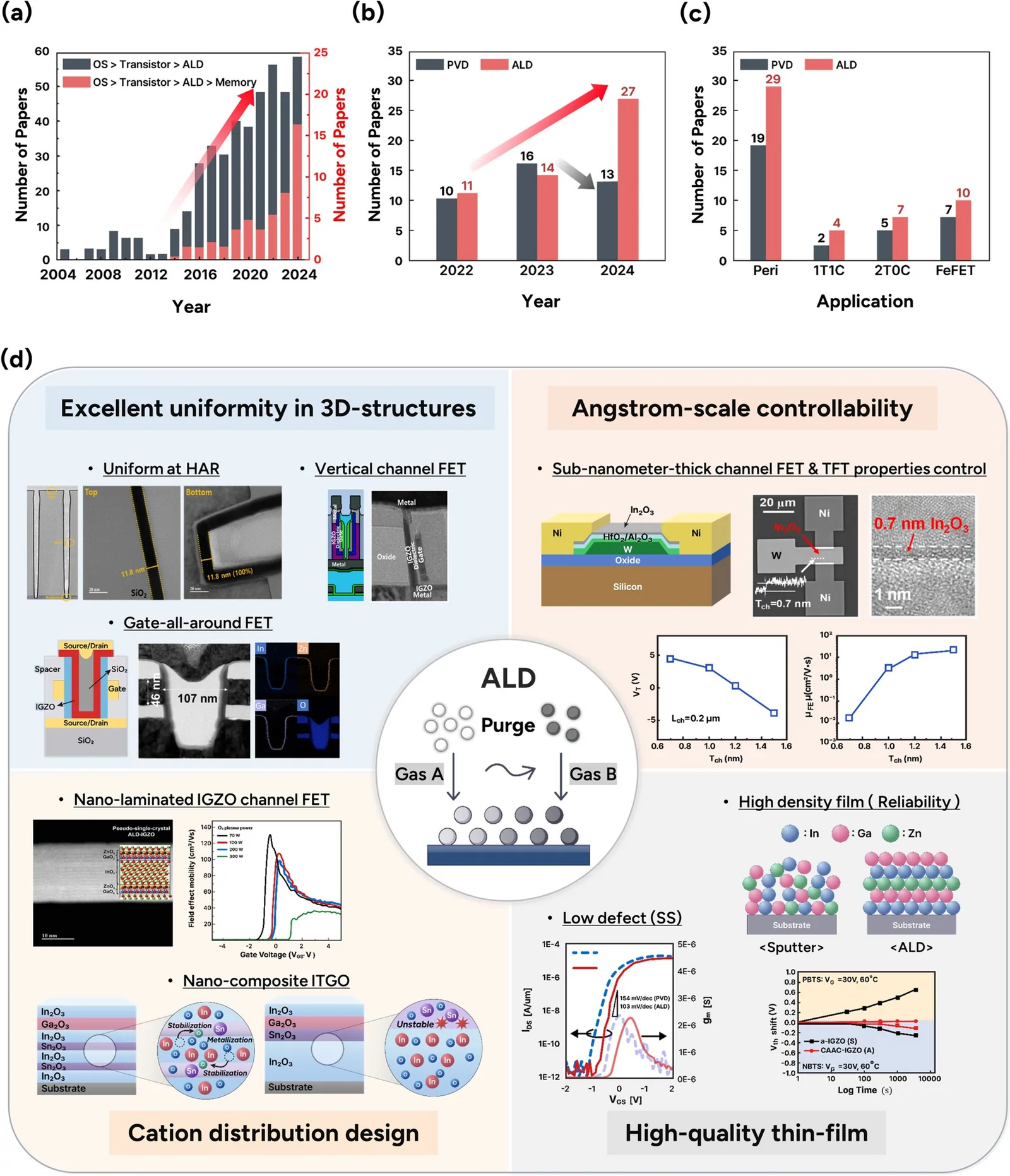
**a** Number of publications from Scopus (search keyword: ALD oxide semiconductor transistor (black column) and memory application (red column)). **b, c** Number of presentations on OSs using PVD and ALD in IMW, VLSI, and IEDM conferences, categorized by year and application. **d** Four key advantages of ALD. Reproduced with permission [34, 61, 125, 128–130]. Copyright 2024, IEEE. Copyright 2020, ACS Publications. Copyright 2022, Springer Nature. Copyright 2023, John Wiley & Sons. Copyright 2024, Elsevier

ALD is essential for the integration of OS channels in memory devices. In conventional display applications, PVD has been sufficient to ensure lateral uniformity over large areas in OS TFTs and has been successfully adopted in mass production. However, to apply OS channels to the highly complex 3D architectures found in memory devices, ALD processes with excellent step coverage are required. As shown in Fig. 4a, the number of publications on ALD-based OS FETs has increased significantly since 2014, reaching approximately 60 papers per year in recent years. This upward trend aligns with the growing interest in applications targeting memory devices, which accounted for more than 25% of all ALD-based OS FET publications in 2024. Figure 4b summarizes the number of OS-related presentations categorized by deposition method at major international conferences (IMW, VLSI, and IEDM) over the past three years. The number of PVD-based studies was 10 in 2022, 16 in 2023, and slightly decreased to 13 in 2024. In contrast, ALD-based approaches exhibited rapid growth, increasing from 11 presentations in 2022 to 27 in 2024. Notably, in 2024, ALD-based presentations outnumbered those based on PVD by approximately twice as many presentations. Furthermore, as shown in Fig. 4c, ALD-based OS channel research outpaces PVD across all memory device categories, including peripheral circuits, 1T1C, 2T0C, and FeFET architectures. Considering these trends, research on ALD-based OS channels for memory applications is expected to show continued growth and interest in the coming years.

As shown in the center of Fig. 4d, ALD is a thin-film growth technique based on the sequential injection of self-limiting gaseous precursors and reactants. The increasing adoption of ALD in OS research for memory applications is attributable to four key characteristics: First, ALD offers excellent uniformity in complex 3D structures. In contrast to PVD, which is limited by the directional nature of vapor transport, ALD achieves conformal coating in complex 3D structures through gas-phase diffusion and self-limiting reactions [34, 124, 125]. Recent emerging 3D DRAM architectures demand conformal deposition techniques in structures with aspect ratios exceeding 20:1. Ryu et al. reported ALD-grown InGaO films (In:Ga = 4:1 at%) exhibiting high mobility (~ 128.2 cm^2^ V^−1^ s^−1^) and thermal stability (~ 700 °C) [126]. To translate this promising channel material into 3D architectures, it is essential to employ a growth technique capable of achieving uniform control over key parameters—such as film thickness, composition, and crystallinity—that critically affect electrical performance. Using ALD, they demonstrated 95% thickness uniformity and less than 1% cation composition variation even in structures with aspect ratios as high as 40:1, while maintaining a uniform crystal structure. These uniform properties of thickness, composition, and crystal structure afford greater flexibility in device architecture design, such as in vertical channel and GAA OS FETs [34, 54, 60, 61].

Second, ALD provides angstrom-level thickness control. The self-limiting nature of the process enables near-monolayer growth per cycle, allowing precise thickness control at the atomic scale [8, 127]. As memory cell sizes continue to scale both laterally and vertically, particularly in vertical channel and GAA structures, thinner channels are required to enhance packing density. Si et al. demonstrated that ALD enables the uniform deposition of In_2_O_3_ channels down to an ultrathin thickness of 0.7 nm [128]. While bulk In_2_O_3_, with its charge neutrality level located approximately 0.4 eV above the conduction band minimum (E_C_), is typically considered a conducting oxide, quantum confinement in channels thinner than 1.5 nm can shift the trap neutral level below E_C_, thereby suppressing the carrier density into the semiconducting regime. As the channel thickness directly influences the threshold voltage, In_2_O_3_ can thus be engineered to exhibit clear switching behavior. Such precise thickness control is essential to ensure uniform device characteristics in densely integrated memory arrays.

Third, ALD enables the design of cation distribution in multicomponent oxides. The electrical properties of OS materials depend strongly on cation distribution, which can be controlled via sub-cycle modulation in ALD. To meet increasing performance demands in memory applications, OS FETs are expected to achieve higher mobilities (> 100 cm^2^ V^−1^ s^−1^) and strong reliability under positive bias-temperature stress (PBTS) at elevated temperatures (e.g., 95 °C). In a prior study, a nano-laminated IGZO channel FET was engineered to incorporate In_2_O_3_, which exhibits high electron conductivity, as the primary conduction path. This design facilitated the formation of multiple 2D electron gas (2DEG) channels, resulting in an enhanced field-effect mobility of approximately 110 cm^2^ V^−1^ s^−1^ [125]. In another study, nano-composite InSnGaO (ITGO) FETs with homogeneously incorporated Sn cations effectively suppressed oxygen vacancies and improved SS (71.9 → 64.8 mV decade^−1^) and PBTS reliability (Δ*V*_th_: + 0.19 → 0.06 V under + 2 MV cm^−1^, 95 °C, 1 h) [129].

Finally, ALD enables the growth of high-quality thin films. PVD inherently induces structural native defects, as the material is physically removed from the target and directly stacked onto the substrate. In contrast, owing to ALD's layer-by-layer film growth based on self-limiting surface chemical reactions, ALD-based OSs forms close-packed films that effectively minimize impurity incorporation and defect formation compared to other deposition techniques. Owing to its layer-by-layer surface reaction mechanism, ALD minimizes impurity incorporation and defect formation compared to other deposition methods. Kim et al. conducted a comparative study of IGZO films (In:Ga:Zn = 1:1:1 at%) deposited by sputtering and ALD [130]. The ALD-grown IGZO exhibited a reduced concentration of oxygen-related defects (from 34.7 to 24.1%) and improved film density (from 6.01 to 6.30 g cm^−3^). These improvements translated directly into enhanced FET performance, with increases in *μ*_FE_ (from 20.5 to 28.1 cm^2^ V^−1^ s^−1^), and reductions in subthreshold swing (from 0.33 to 0.23 V decade^−1^) and hysteresis (from 0.17 to 0.04 V). Similar improvements in both material and electrical properties have also been reported for other ALD-deposited thin films compared to their PVD counterparts [34, 131]. Given these advantages, research on ALD-based OSs has been actively expanding, with increasing efforts focused on controlling ALD process parameters to optimize electrical performance.

### Engineering OSs based on ALD Fundamentals: Adsorption & Reaction

**Fig. 5:**
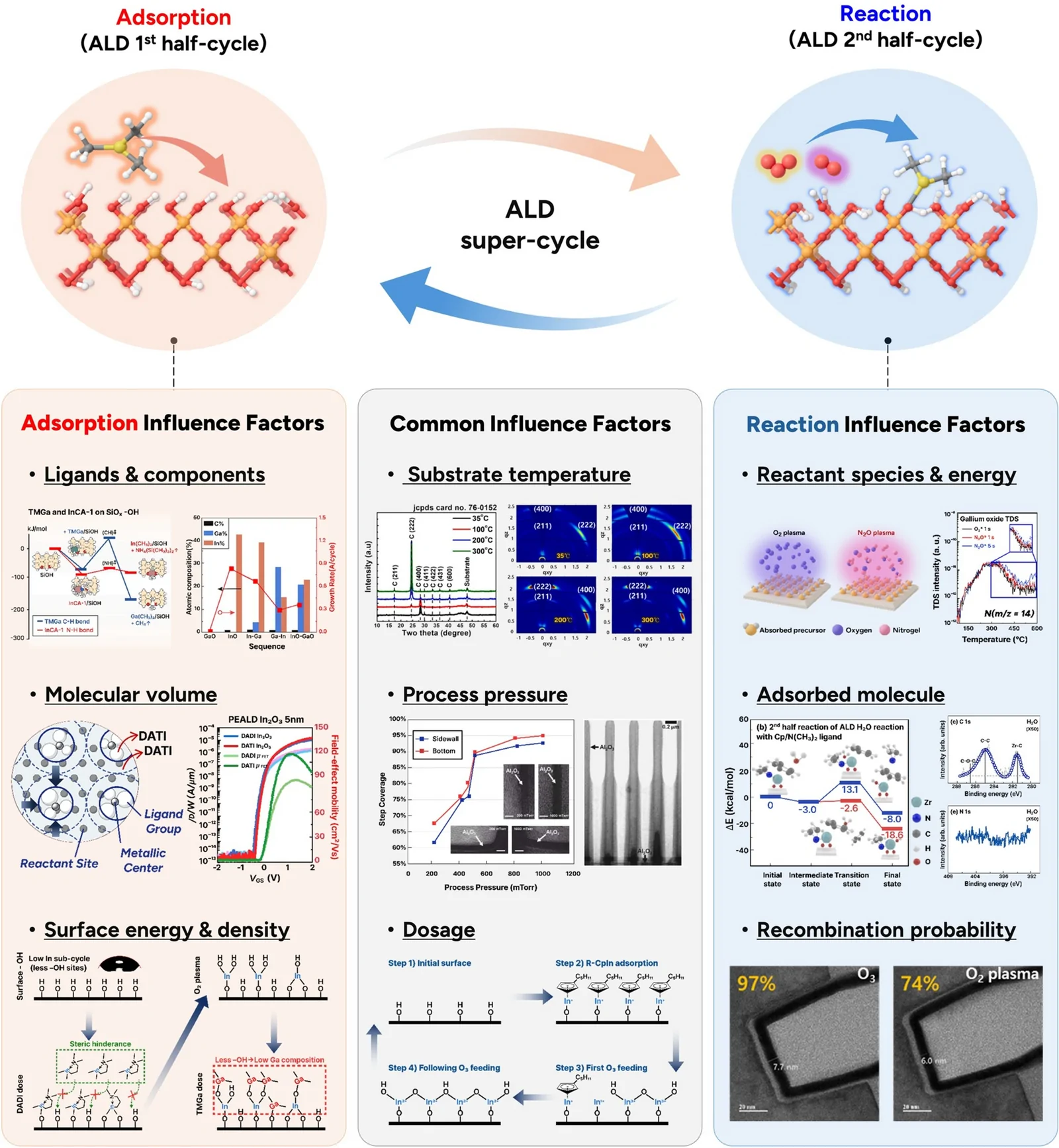
Overview of ALD process parameters for Oxide Semiconductors: Factors influencing the adsorption step, reaction step, and both adsorption and reaction. Reproduced with permission [132, 147, 149, 177, 192, 193, 200, 210]. Copyright 2017, 2021, 2023 ACS Publications. Copyright 2022, 2023, 2024, Elsevier. Copyright 2007, The Electrochemical Society

The fundamental principle of ALD pertains to self-limiting chemical reactions, which occur in two key steps: surface adsorption and reaction, as illustrated in Fig. 5. Various process parameters affect each step of the cycle, ultimately determining the properties of the resulting oxide film. Therefore, a thorough understanding of ALD process variables and their influence on surface reaction behavior is essential for engineering OS channels.

#### Factors Affecting Adsorption (1st Half-Cycle) for OSs Engineering

The first half-cycle of ALD, adsorption, involves the interaction between the precursor and reactive sites on the surface, resulting in the formation of chemisorbed species. This step is the primary consideration when designing both the process and the film properties. As illustrated in the adsorption influence factors shown in Fig. 5, several key parameters affect this process. First, the types of precursor ligands and metal components play a critical role. The adsorption energy (Δ*E*_ads_) and activation energy (*E*_a_) of the surface reaction vary depending on the metal center and ligand structure of the precursor. These factors determine whether adsorption occurs and which pathway is preferred. As adsorption reactions are generally associated with a reduction in entropy (Δ*S* < 0), the spontaneity of the reaction can be assessed based on the sign of Δ*E*_ads_: A negative Δ*E*_ads_ indicates a spontaneous process, while a positive value suggests a nonspontaneous reaction, providing insights into the suitability of a precursor for ALD applications [132, 133]. Among multiple spontaneous adsorption pathways, the one with the lowest *E*_a_ dominates, and this information can help predict both the reaction mechanism and configuration of the adsorbed species [134, 135]. Sheng et al. employed density functional theory to calculate the adsorption energy profiles of an In precursor, diethyl[1,1,1-trimethyl-N-(trimethylsilyl)-silanaminato]-indium (InCA-1), and a Ga precursor, trimethylgallium (TMGa), on a SiO_2_ substrate [132]. Both precursors exhibited negative adsorption energies—Δ*E*_ads_ =  − 90.6 kJ mol^−1^ for InCA-1 and − 72.1 kJ mol^−1^ for TMGa—indicating favorable adsorption for ALD. However, the *E*_a_ for TMGa was significantly higher (~ 100 kJ mol^−1^) compared to that of InCA-1 (14.5 kJ mol^−1^), suggesting a kinetic limitation that hinders initial growth. These findings highlight the importance of precursor-specific kinetics and were utilized to optimize sub-cycle sequencing for effective film growth and compositional control in multicomponent IGO systems. *E*_a_ also defines the energy barrier: an increase in substrate temperature leads to a higher reaction probability and improves reaction completion. Thus, *E*_a_ ultimately determines the minimum substrate temperature at which adsorption begins [136]. When the second half-cycle is sufficiently reactive, the ALD temperature window can be affected by the ligand structure of precursors, as summarized in Table 3 [137–175]. Choi et al. reported that an amine ligand-based In precursor, dimethyl[N-(tert-butyl)-2-methoxy-2-methylpropan-1-amine] indium (DMION), enables a reduction of the ALD window lower limit to 35 °C, compared to conventional alkyl ligand-based In precursors [149]. This low-temperature process yielded high-quality, carbon-free (< 0.1 at%) films, and the resulting material demonstrated viable FET operation, with a *V*_th_ of 4.9 V and *μ*_FE_ of 3.1 cm^2^ V^−1^ s^−1^. These results indicate that the target process temperature can be tailored by modifying the ligand structure. In addition, precursor behaviors such as decomposition, condensation, and desorption must be carefully considered for optimal process design.

**Table 3:** ALD process information for InO_x_, GaO_x_, ZnO_x_, and SnO_x_, and the material properties

	Precursor	Reactant	Growth Temperature (°C)	GPC (A˚ cycle^ − 1^)	Impurity 1) (%)	*E*_*g*_ (eV)	Crystallinity	Hall mobility (cm^2^ V ^− 1^ s^ − 1^)	TFT mobility (cm2 V − 1 s − 1)	Refs
Indium oxide	TMIn	H_2_ O/O_2_	100–250	0.2–1.6	–	–	–	38–111	–	[137]
	TMIn	Ozone	100–200	0.46	C ~ 1.5 at%	3.6	–	∼50	–	[138]
	TMIn	Ozone	100–250	0.6	–	3.8	Cubic > 200 °C	–	–	[139]
	TMIn	H_2_O/O_2_	100–250	1.5	–	–	Cubic > 200 °C	∼49	–	[140]
	TMIn	Ozone	200–450	0.3–2.0	–	–	Cubic > 140 °C	–	–	[141]
	TMIn	Ar/O_2_ radical	100–400	0.14	C	3.6	Cubic at 275 °C	–	–	[142]
	TMIn	H_2_O	165–200	0.2	–	–	Cubic > 100 °C	–	–	[143]
	TMIn	Ozone	165–225	0.12	–	–	–	–	–	[143]
	TMIn	H_2_O	150–250	0.1–0.3	N/D	3.7	–	∼41	–	[144]
	TEIn	Ozone	100–250	0.8	–	3.9	Cubic > 200 °C	–	–	[139]
	InCp	H_2_O	175–275	0.7	Si, C	3.9	Cubic > 200 °C	–	–	–
	–	H_2_O_2_	125–225	0.7	Si, C	4	Cubic > 200 °C	–	∼15	[145]
	In(tmhd)^3^	Ozone	150–300	0.7	N/D	3.6	Cubic > 200 °C	∼51.8	–	[139]
	In(acac)^3^	H_2_O	> 275	0.6	$\text{C > 1 at%}$	3.6	Cubic > 200 °C	–	–	[146]
	Me_2_In(edpa)	Ar/O_2_ plasma	100–250	0.8	N/D	3	Cubic > 275 °C	–	∼10	[147]
	InCA − 1^2^)	H_2_O	100–250	0.2	C	3	Cubic > 100 °C	∼1.0	∼6.1	[148]
	DADI^3^	–	–	–	–	–	Rhombohedral < 200 °C	–	–	–
	–	–	–	–	–	–	Cubic > 200 °C	–	–	–
	TMION^4^	Ar/O_2_ plasma	100–250	1.1	N/D	3.4	Cubic > 100 °C	∼5.1	∼20.6	[147]
	–	Ar/O_2_ plasma	35–300	1.2	N/D	3.4	Cubic > 35 °C	∼5 < 100∘C	∼3.1 < 100∘C	[149]
	DMION^4^	–	–	–	–	–	–	∼33 > 100∘C	∼34.4 > 100∘C	–
	DMITN^4^	H_2_O	200–250	0.3	N/D	3.3	Cubic > 200 °C	∼2	–	[150]
	DATI^3^	Ar/O_2_ plasma	100–250	1.1	N/D	3.5	Cubic > 100 °C	∼9	–	[150]
	Indium oxide	Ar/O_2_ plasma	100–250	1	N/D	3.4	Cubic > 100 °C	∼1.1	∼17.5 > 250∘C	[151]
Tin	TDMASn	H_2_O	30–200	0.3–2.0	N/D	3.6–4.0	Tetragonal	–	–	[166]
oxide	BDMADMSn	Ozone	50–250	0.98–1.60	N/D	2.9–4.0	Tetragonal	~ 32	–	[167]
		Ar/O_2_ plasma	100–250	~ 1.25	N/D	3.6	Tetragonal	–	–	[168]
	SnCl_4_	Ozone	150–250	~ 0.49	N/D	–	Tetragonal	~ 2.7	–	[169]
	HS-	H_2_O_2_	250	1.22–1.38	–	3.6	Tetragonal	~ 9.9	–	[170]
	Stannylene-01	H_2_O	60–150	0.1–0.5	N/D	~ 1.6	Tetragonal (> 100 °C)	~ 18	~ 6.0 (p-type)	[172]
		Ozone	80–250	~ 1.4	N/D	~ 4.1	Tetragonal	~ 24	–	[172]
	Sn(edpa)^2^	Ar/O_2_ plasma	60–300	0.2	N/D	~ 2.9	–	–	–	[173]
		H_2_O	60–175	0.7	N/D	~ 4.0	Tetragonal (> 70 °C)	~ 27	–	[173]
	Sn(dmamp)^2^	H_2_O	175–275	–	N/D	–	Polycrystalline	–	~ 7.24	[174]
		Ozone	100–230	0.18–0.42	N/D	3.6	Tetragonal	–	–	[175]
Gallium	Ga(acac)^3^​	Ozone	150–300	0.16–0.54	N/D	4.5	–	–	–	[152]
oxide	TMGON	Ar/O2​ plasma	150–250	0.75–1.30	N/D	4.6–4.8	–	–	–	[147]
	DMGON	Ozone	250	0.62	N/D	4.5	–	–	–	[153]
	DMGTN	Ar/O_2_ plasma	365–380	0.33	–	–	–	–	–	[154]
	GTIP	Ozone	350–375	0.22	–	–	–	–	–	[154]
	Ga(tmhd)^3^	Ar/O_2_ plasma	100–250	1.05	C∼1.2 at%	–	–	–	–	[146]
	Ga_2_(NMe_2_)^6^	Ozone	150–300	0.11–0.46	N/D	–	–	–	–	[126]
	GaI_3_	Ar/O_2_​ plasma	100–250	0.92–1.32	N/D	5.2	–	–	–	
	TEGa	H_2_O	150–300	0.10–0.40	N/D	–	–	–	–	
	TMGa	Ar/O_2_ plasma	100–400	1.17	N/D	5.2	–	–	–	[155]
		H_2_O	150–250	2.5	N/D	~ 5.4	–	–	–	[156]
	DEZ	Ar/O_2_ plasma	60–160	0.1	N/D	5	–	–	–	[157]
		Ozone	150–550	0.9–1.1	C∼2.1 at%	–	–	–	–	[158]
		H_2_O_2_	100–200	1.5	I∼3.2 at%	–	–	–	–	[159]
		Ozone	100–200	0.5–1.75	–	4.9	–	–	–	[160]
		H_2_O	150–300	~ 1.50	N/D	3.2	Hexagonal	~ 10	–	[160]
		Ar/O_2_ plasma	100–200	~ 1.58	N/D	3.2	Hexagonal	~ 11	–	[161]
		Ozone	150–300	~ 1.61	N/D	3.2	Hexagonal	~ 3.0	–	[162]
	Zn(DMP)_2_	Ar/O_2_ plasma	100–250	1.57–1.70	(> 200 °C)	3.2	Hexagonal	–	–	[163]
	DEZDMEA	H_2_O	150–250	0.9	N/D	3.2	Hexagonal	–	0.04	[164]
		H_2_O	140–180	0.7–1.1	N/D	3.2	Hexagonal	–	–	[165]
		H_2_O	50–250	2.56	N/D	3.2	Hexagonal	–	–	[165]

^1^Impurity level is estimated by XPS. (Limit of detection: < 0.1 at%)

^2^^−^^4^Provided by 2) UP-Chemical, 3) LAKE Materials, 4) Hansol Chemical

Second, the molecular volume of the precursor considerably affects adsorption. Even if a certain adsorption pathway is thermodynamically favored, steric hindrance can prevent the precursor from accessing all surface reactive sites. As the volume of the adsorbed species increases, steric limitations become more severe, which reduces the number of precursor molecules that can adsorb per cycle, resulting in a lower growth per cycle (GPC). For example, Oh et al. compared the ALD behavior of trimethylaluminum (TMA) and triethylaluminum (TEA) based on their molecular sizes [176]. The effective average molecular volumes were 87.2 Å^3^ for TMA and 140.2 Å^3^ for TEA, with the larger TEA molecule exhibiting a reduced GPC of 0.8 Å cycle^−1^, compared to 1.2 Å cycle^−1^ for TMA. Similarly, in the ALD of crystalline In_2_O_3_, the use of a smaller-volume precursor, (N,N-dimethylbutylamine)trimethylindium (DATI), led to enhanced adsorption coverage, resulting in improved film density (6.57 → 6.76 g cm^−3^), crystallinity, and* μ*_FE_ (90.5 → 115.8 cm^2^ V^−1^ s^−1^), compared to a larger precursor, (3-(dimethylamino)propyl)-dimethyl indium (DADI) [177]. In multicomponent systems, larger dopant precursors have been found to promote more homogeneous doping. For example, homogeneous cation distributions in crystalline InGaO improve grain alignment and *μ*_FE_ (~ 128.2 cm^2^ V^−1^ s^−1^) upon crystallization [126], while in amorphous AlZnO systems, reduced electron scattering leads to enhanced conductivity [178]. These findings suggest that controlling adsorption behavior through molecular volume design of precursors is an effective strategy for tuning the structural and electrical properties of thin films [126, 176–178].

Finally, surface energy and the density of reactive sites also strongly influence adsorption. Surface energy depends on various factors, such as substrate type and orientation, material deposited in the previous cycle, and terminal surface functional groups. High surface energy generally promotes reactivity by lowering the adsorption energy, whereas low surface energy suppresses film growth. Reactive site density is influenced by surface roughness, film density, and material composition. A high density of reactive sites facilitates greater adsorption coverage. Studies have demonstrated that controlling the surface condition based on appropriately selecting the substrate, seed layers, material deposited in the previous ALD cycle, or surface treatments can effectively tune the ALD temperature window, growth rate, crystallinity, electrical properties, and composition controllability [147, 179–187]. Notably, Hong et al. showed that the In/Ga ratio and electrical properties of InGaO films varied depending on the precursor pairing. trimethyl[N-(2-methoxyethyl)-2-methylpropan-2-amine] indium (TMION)/trimethyl[N-(2-methoxyethyl)-2-methylpropan-2-amine]gallium (TMGON) pairing enabled linear composition control due to matched adsorption characteristics, while DADI/TMGa showed Ga-rich deviation from enhanced –OH surface reactivity. Consequently, *μ*_FE_ reached 36.7 cm^2^ V^−1^ s^−1^ (TMION/TMGON-IGO) and 27.7 cm^2^ V^−1^ s^−1^ (DADI/TMGa-IGO) at an equivalent In/Ga ≈ 2.3 ratio. These findings highlight that surface conditions play critical roles in determining composition and growth behavior in multicomponent ALD [147, 179–187]. In summary, the adsorption step is critically influenced by both precursor characteristics and surface conditions, and it plays a central role in determining the adsorption mechanism, process temperature, and resulting film properties.

#### Factors Affecting Reaction (2nd Half-Cycle) for OSs Engineering

In the second half-cycle of ALD, referred to as the reaction step, the injected reactant reacts with the ligands of the adsorbed precursor, leading to ligand removal and the regeneration of reactive sites on the surface. This step completes the film formation process. As illustrated in the reaction influence factors in Fig. 5, several parameters affect the reaction process. First, the type and energy of the reactant play a crucial role. In oxide deposition, typical oxygen sources include H_2_O, H_2_O_2_, O_2_, O_3_, O_2_ plasma, and N_2_O plasma. The reaction mechanisms vary depending on the type of reactant, proceeding either through ligand exchange or combustion. H_2_O, which follows a ligand exchange mechanism, was widely used in early ALD processes owing to its high vapor pressure and environmental friendliness. However, its low oxidation strength limits the process temperature window and can lead to residual hydrogen impurities, necessitating alternative oxygen sources. Stronger oxidants such as H_2_O_2_, O_3_, and O_2_ plasma, all of which follow combustion mechanisms, have been employed to address these limitations. The relative oxidation strength follows the order H_2_O < H_2_O_2_ < O_3_ < O_2_ plasma [150, 188–190]. Increased oxidation strength is generally associated with a wider ALD temperature window and higher GPC. Furthermore, stronger oxidants reduce impurity incorporation and defect density, improving film crystallinity and electrical properties. According to Ryu et al., ALD using H_2_O as the reactant exhibited a growth per cycle (GPC) of 0.29 Å cycle^−1^ within an ALD window of 200–250 °C, whereas the use of O_2_ plasma extended the ALD window to 100–250 °C and significantly increased the GPC to 1.11 Å cycle^−1^ [150]. In_2_O_3_ films grown with O_2_ plasma demonstrated a high film density (~ 7.0 g cm^−3^), reduced oxygen-related defects, and a low crystallization temperature (< 100 °C. Moreover, as indicated in Table 3, a comparison of Ga precursors, TMGa, dimethyl[N-(tert-butyl)-2-methoxy-2-methylpropan-1-amine] gallium (DMGON), and dimethyl[N1-(tert-butyl)-N2,N2-dimethylethane-1,2diamine] gallium (DMGTN), and the Sn precursor, bis(dimethylamino) dimethyltin (BDMADMSn), shows that using O_2_ plasma—which has a higher oxidation energy than O_3_—tends to increase the GPC by nearly a factor of three. In the case of Sn-based oxides, oxidation energy can be tuned to selectively form either p-type SnO or n-type SnO_2_. Lee et al. demonstrated that SnO and SnO_2_ phases could be selectively synthesized via ALD by simply switching the oxidant: water favored SnO formation with a bandgap of ~ 2.3 eV, while ozone induced SnO_2_ growth with a bandgap of ~ 4.0 eV. This phase control was enabled by the differing oxidative strengths and surface reactivities of H_2_O and O_3_ [172]. O_2_ plasma offers the additional advantage of high oxidation strength along with tunable reactivity through plasma power control. However, it may also induce surface damage owing to the presence of energetic Ar radicals, necessitating careful optimization of process conditions to balance oxidation efficacy with surface integrity [125, 150, 191]. N_2_O plasma has also been investigated owing to its strong reactivity and additional benefit of nitrogen incorporation, which can enhance device reliability [192]. According to Kim et al., selective introduction of an N_2_O plasma reactant during the Ga_2_O_3_ sub-cycle of the IGZO process enabled nitrogen doping at a concentration of 0.2 at%, which preserved high *μ*_FE_ (~ 106.5 cm^2^ V^−1^ s^−1^) while simultaneously achieving excellent PBTS reliability (Δ*V*_th_: + 0.45 V under + 2 MV cm^−1^, 95 °C, 10,000 s). These findings underscore the significance of oxidant selection as a versatile tool for precisely controlling film properties, including composition, crystallinity, electrical performance, and dopant incorporation, in oxide thin-film deposition.

Second, the type and structure of the adsorbed precursor species significantly influence the reaction. Even when the reactant is highly reactive, the reaction energy (Δ*E*) and *E*_a_ can vary depending on the metal center and ligand configuration of the adsorbed molecule. These energy parameters affect both the reaction efficiency and the ALD temperature window. As indicated in Table 3, different metal–ligand combinations require distinct thermal and oxidative energy inputs during ALD. A comparison between trimethylindium (TMIn) and TMGa—precursors with the same ligand structure—under O_3_ reactant conditions reveals notable differences in growth behavior. While TMIn exhibits a relatively constant GPC of ~ 0.46 Å cycle^−1^, the GPC of TMGa increases from 0.16 to 0.54 Å cycle^−1^ with rising process temperature. This trend suggests that Ga-centered adsorption molecules require higher oxidation energy compared to their In-based counterparts, implying that insufficient energy supply during the ALD process may lead to a reduced GPC or the formation of higher impurity-containing films [193]. Therefore, the reactant must be carefully matched to the precursor and intended process temperature.

Finally, the recombination probability of the reactant affects film conformality, especially in 3D memory structures. Even when a reactant is thermodynamically and kinetically favorable, its effective diffusion length must be sufficient to ensure uniform deposition across high-aspect-ratio features. Reactants with high recombination probabilities may deplete rapidly near the feature opening, leading to thicker deposition near the top and thinner coverage at the bottom, a phenomenon known as the recombination-limited regime [194]. For example, oxygen radicals used in plasma-enhanced ALD exhibit high recombination rates, limiting their ability to achieve ideal conformality compared with thermal ALD processes [150, 195–197]. According to Ryu et al., In_2_O_3_ films grown using H_2_O or O_3_ exhibited excellent conformality in structures with a 40:1 aspect ratio, achieving bottom-step coverage of 97% and sidewall-step coverage of 95%. In contrast, when O_2_ plasma was used, the bottom-step coverage decreased to 74%, indicating a limitation in achieving conformal deposition [150]. In summary, the reaction step is strongly influenced by both the reactant properties and the structure of the adsorbed species. These factors collectively determine the process temperature, film quality, and step coverage in ALD-grown OSs.

#### Common Influencing Factors for OSs Engineering

Certain factors influence both the adsorption and reaction steps. As shown in Fig. 5, the three representative process parameters are temperature, pressure, and dosage. First, substrate temperature plays a critical role. Increasing the process temperature enhances the probability of overcoming the energy barriers associated with adsorption and reaction, promoting reaction completeness. Elevated temperatures have been reported to reduce impurities and defects, increase film density and crystallinity, and improve electrical performance across various OS materials [134, 149, 150, 198, 199]. For example, in the In_2_O_3_ study reported by Ryu et al., increasing the process temperature from 100 to 250 °C—regardless of the reactant type—led to a decrease in carbon impurity (< 0.1 at%), reduction in oxygen-related defect bonding (~ 20.0%), increase in film density (~ 7.0 g cm^−3^), enhanced orientation along the cubic (222) plane, and improved Hall mobility (~ 38.8 cm^2^ V^−1^ s^−1^), demonstrating the significant influence of temperature [150]. However, the process temperature must remain below the precursor decomposition or desorption threshold to preserve the self-limiting behavior of ALD.

Second, process pressure significantly influences the frequency of molecular collisions with the substrate. At low pressures, reduced collision frequency may lead to longer process times, increased precursor consumption, and poor step coverage in high-aspect-ratio structures. Conversely, higher pressure can alleviate steric hindrance and enhance diffusion to reactive sites, resulting in more uniform coverage and improved film quality [200, 201]. According to Li et al., increasing the process pressure from 200 to 1000 mTorr at a fixed process temperature led to a steady rise in the GPC of Al_2_O_3_ from 1.01 to 1.05 Å cycle^−1^. Concurrently, improvements in insulating properties, such as higher breakdown voltage and reduced leakage current, were also observed. Recently, atmospheric pressure processes utilizing spatial ALD have been reported, offering advantages in throughput and precursor utilization [202–205]. According to Yoo et al., the deposition rate of In_2_O_3_ was significantly enhanced to 30.0 Å min^−1^, compared to the conventional ALD process rate of approximately 2.5 Å min^−1^ [202]. However, these approaches present challenges related to purge efficiency and potential contamination [206].

Finally, precursor and reactant dosages must be sufficient to ensure saturation of surface sites during both adsorption and reaction. Dosage is typically controlled by adjusting pulse duration or canister temperature. Saturation is typically determined based on the condition where the GPC reaches a constant value. In standard single-dose ALD processes, physisorbed molecules may block reactive sites, preventing complete saturation. This limitation can be addressed using discrete feeding method (DFM), in which an initial purge is followed by a second pulse to achieve denser precursor coverage. This technique has been shown to improve film density, enhance crystallinity, and reduce impurities [207–209]. Kim et al. introduced a DFM precursor into the p-type SnO process to enhance the formation of a (001)-aligned tetragonal 2D structure. As a result, the fabricated SnO-FETs exhibited improved performance with a *μ*_FE_ of 1.86 cm^2^ V^−1^ s^−1^, a SS of 0.12 V decade^−1^, and stable PBTS reliability (Δ*V*_th_: + 0.47 V under + 2 MV cm^−1^, 60 °C, 10,000 s) [207]. Additionally, in cases where precursors with bulky cyclopentadienyl ligands are employed, applying a discrete reactant feed (DRF) has been reported to enhance reaction completeness [210]. According to Yang et al., the use of a DRF approach enhanced the reactivity of a Cp ligand-based In precursor, increasing the GPC to 2.2 Å cycle^−1^ compared to 1.3 Å cycle^−1^ achieved by the conventional method. By carefully controlling these ALD process parameters, OS films that meet the stringent requirements of memory device applications can be engineered.

### Advanced Developed ALD-Driven OSs via Four Main Material Design Strategies

Recent studies on ALD-driven OSs for memory applications have focused on four main material design themes—cation engineering, crystallization control, atomic structure optimization, and light element incorporation—to enhance electrical performance, reliability, and thermal stability.

#### Cation Engineering

The cations that constitute the matrix of OSs represent a primary focus in material design. In OS channels, metal cations significantly influence key material properties, such as charge transport, defect formation, and structural stability. Electronic structure of the cations affects the dispersion of the conduction band, thereby impacting charge transport property. Their bonding strength with oxygen influences the subgap defect states, which are closely linked to device reliability. In addition, the metal cation can affect the stability of the structural properties, compatibility with temperature dependent processing. As these factors collectively determine the electrical performance and long-term stability of OS channels, precise cation engineering is essential for material optimization [117, 120].

**Fig. 6:**
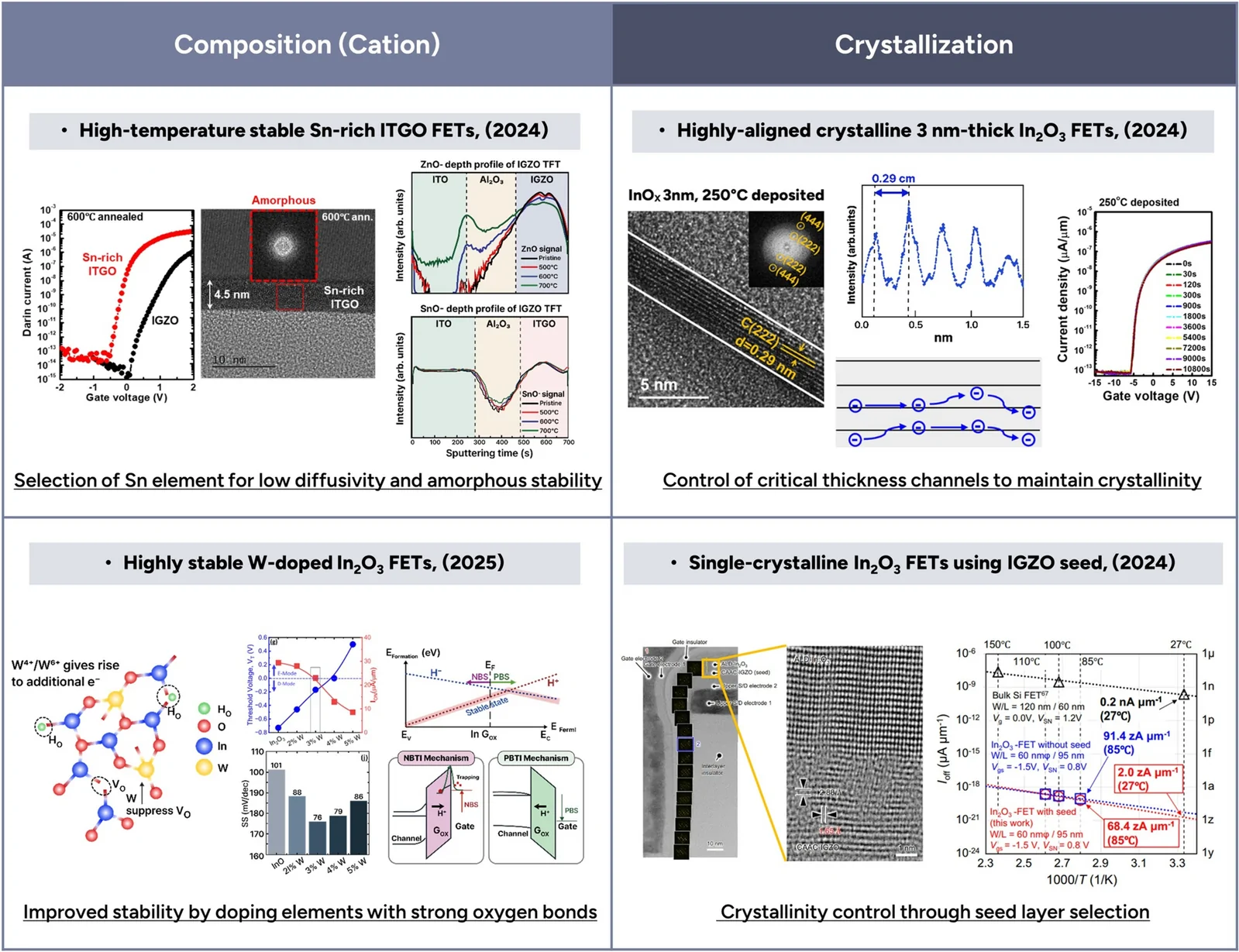
Recent strategies for enhancing the electrical properties and reliability of oxide semiconductor FETs through cation engineering (left) and crystallinity control (right). Reproduced with permission [169, 212, 215, 218]. Copyright 2024, ACS Publications. Copyright 2025, IEEE. Copyright 2024, Nature

As shown in the left part of Fig. 6, one representative approach involves incorporating cations such as Sn and W, which exhibit metal–oxygen bond dissociation energies of 548 and 653 kJ mol^−1^, respectively, into In-based OSs to strengthen metal–oxygen bonding [211]. Through their precise introduction at controlled concentrations using ALD, the electrical characteristics, reliability, and thermal robustness of OSs can be enhanced. In terms of the introduction of Sn, Ryu et al. [169] demonstrated that a Sn-rich ITGO amorphous OS developed via ALD exhibits exceptional thermal stability and electronic performance suitable for DRAM applications. The optimized ITGO composition (In:Sn:Ga = 25:58:17 at%) maintained an amorphous structure and exhibited high Hall mobility (~ 24.0 cm^2^ V^−1^ s^−1^) even after annealing at 600–700 °C. Sn incorporation stabilized the amorphous phase, reduced oxygen vacancies, and enhanced carrier mobility, while Ga suppressed excessive carrier generation and preserved thermal stability. A 4.5-nm-thick ITGO TFT showed excellent field-effect mobility (7.7 cm^2^ V^−1^ s^−1^) and remarkable bias-temperature reliability (Δ*V*_th_: − 0.05 V under PBTS and + 0.01 V under negative bias-temperature stress (NBTS) at 125 °C for 2000 s) [169].

In terms of the incorporation of W, Sarkar et al. [212] reported the first GAA nanosheet FET incorporating an ALD-IWO channel, which exhibited excellent scalability and electrical reliability. The small ionic radius of W allowed for stable substitution into the In_2_O_3_ lattice without significant lattice distortion, contributing to structural integrity and defect suppression. For fabricating the GAA FET, a novel channel release method using a W sacrificial layer enabled lithography-independent definition of the channel length with etch selectivity > 10^3^, simplifying fabrication. The optimized 3% W-doped IWO nanosheet device demonstrated a high on-state current (815 µA µm^−1^ at *V*_ds_ = 1 V) and ultralow off-state current (3 fA), alongside an intrinsic transconductance of 470 µS µm^−1^. It achieved record-low threshold voltage shifts (Δ*V*_th_ ≈ 88 mV at 5.4 MV cm^−1^) under both positive and negative BTI conditions, outperforming existing AOS-based FETs. These results establish ALD-IWO GAA FETs as a promising candidate for BEOL-compatible, high-performance 3D integrated memory and logic applications [212].

In addition, with respect to W incorporation into In_2_O_3_-based channels, Chiang et al. (2025) report the first BEOL-compatible ALD IWTO TFTs with an ultrathin 2 nm channel and L_CH_ down to 70 nm. The devices exhibit enhancement-mode operation with *I*_on_/*I*_off_ > 10^10^, SS ≈ 63 mV dec^−1^, DIBL ≈ 37.8 mV V^−1^, and low contact resistance (≈ 0.72–0.86 kΩ µm) after O_2_ annealing. These results show that high-BDE W/Sn co-doping combined with a ≤ 350 °C O_2_ anneal mitigates short-channel *V*_th_ roll-off and the *I*_on_–*V*_th_ trade-off, positioning IWTO as a strong candidate for monolithic 3D BEOL integration [213]. About other cation of Al, Ding et al. [214] reported fully ALD-fabricated InAlO TFTs, establishing an ALD-only route to composition-tuned In_2_O_3_ channels. The study delivers a balanced performance/stability window—μ ≈ 7.2 cm^2^ V^−1^ s^−1^, SS ≈ 165 mV dec^−1^, *I*_on_/*I*_off_ ≈ 2.3 × 10^6^, *V*_th_ ≈ 0.1 V, and Δ*V*_th_ ≈ 0.11 V under PBS—achieved at a 350 °C anneal. They attribute the gains to ALD-enabled Al incorporation that suppresses oxygen-vacancy formation, highlighting a scalable, low-temperature pathway for next-generation oxide FETs [214]. Collectively, these findings underscore the effectiveness of incorporating high-bond-energy cations via ALD in enabling thermally robust and electrically reliable OSs suitable for next-generation memory and logic device integration.

#### Crystallization Control

Crystallization plays a critical role in enhancing both mobility and stability of oxide semiconductor (OS) channels. A periodic lattice structure enables effective overlap of extended s-orbitals, thereby facilitating high carrier transport. Among the factors that degrade the stability of OSs, those associated with structural disorder include interstitial metal atoms, antisite defects in multi-cation OSs, and grain boundaries (GBs) in polycrystalline films [126, 215–217]. Among these, numerous studies have reported that enhancing the crystallinity of OSs—thereby reducing GB density—effectively improves stability. GBs generally act as charge-trap-rich defective regions that induce carrier scattering, and promote *V*_th_ shift under bias temperature stress. Consequently, randomly oriented crystallization (large mosaic spread with small grains) produces a high areal GB density and abundant GB trap states, degrading mobility and reliability, whereas highly aligned crystalline texture (preferred orientation) suppresses GB density and suppress carrier scattering. In parallel, increasing grain size reduces total GB area per unit volume, lowering GB-mediated trapping. Compared to amorphous counterparts, crystallized films exhibit reduced defect densities—including oxygen vacancies and structural disorders; this reduction suppresses charge trapping and improves bias-stress stability. Furthermore, crystalline OS channels have recently been recognized for their structural robustness, which contributes to enhanced thermal and electrical durability, thereby improving their compatibility with high-temperature processes. As such, controlling crystallinity—with emphasis on orientation control and grain-size enlargement to minimize GBs—has become a key strategy for developing high-performance and reliable OS channel materials [5, 33, 126, 215].

As shown in the right part of Fig. 6, ALD has been used to achieve highly oriented crystallization in ultrathin films on the nanometer scale, as well as highly uniform crystallinity throughout complex structures. In terms of achieving highly oriented crystallization, Choi et al. (2024) demonstrated the development of highly crystalline 3-nm-thick In_2_O_3_ films via ALD using a novel liquid indium precursor, (N,N-di-tert-butylacetimidamido)dimethylindium (DBADMIn). At an optimized deposition temperature of 250 °C, the In_2_O_3_ films exhibited strong C-axis aligned C(222) orientation, resulting in enhanced film quality with reduced grain boundary scattering. The fabricated TFT demonstrated high field-effect mobility (41.12 cm^2^ V^−1^ s^−1^), low SS (150 mV dec^−1^), and excellent positive bias stress stability (Δ*V*_th_ =  + 0.16 V at 100 °C for 3 h). The improvement in performance was attributable not to oxygen vacancy modulation but to crystalline orientation control, overcoming the traditional mobility–stability trade-off. These findings highlight the potential of low-temperature, crystalline In_2_O_3_ for highly scaled logic and memory devices [215].

In terms of achieving uniform crystallization over complex structures, Yamazaki et al. [218] fabricated a vertical FET using single-crystalline In_2_O_3_ channels grown by ALD on an insulating film, enabled by a 2-nm-thick CAAC-IGZO seed layer. The single-crystalline In_2_O_3_ films exhibited uniform (111) orientation with no grain boundaries along the current path, achieving a high on-state current (28.8 µA), low threshold voltage variation (σ = 0.05 V), and steep subthreshold slope (86.7 mV dec^−1^). Importantly, the off-state current was reduced to an ultralow value of 2.0 zA µm^−1^ at 27 °C—10 orders of magnitude lower than that of bulk Si FETs. These improvements were attributable to solid-phase epitaxial growth, facilitated by atomic alignment between the CAAC-IGZO (001) plane and In_2_O_3_ (111) plane. This architecture enabled 3D monolithic integration with energy-efficient logic design, offering a scalable solution for next-generation AI and server hardware [218]. Together, these advancements demonstrate that ALD-driven control of crystalline orientation and uniformity in ultrathin oxide films is critical for achieving high-performance, low-power logic and memory devices.

#### Atomic Structure Optimization

**Fig. 7:**
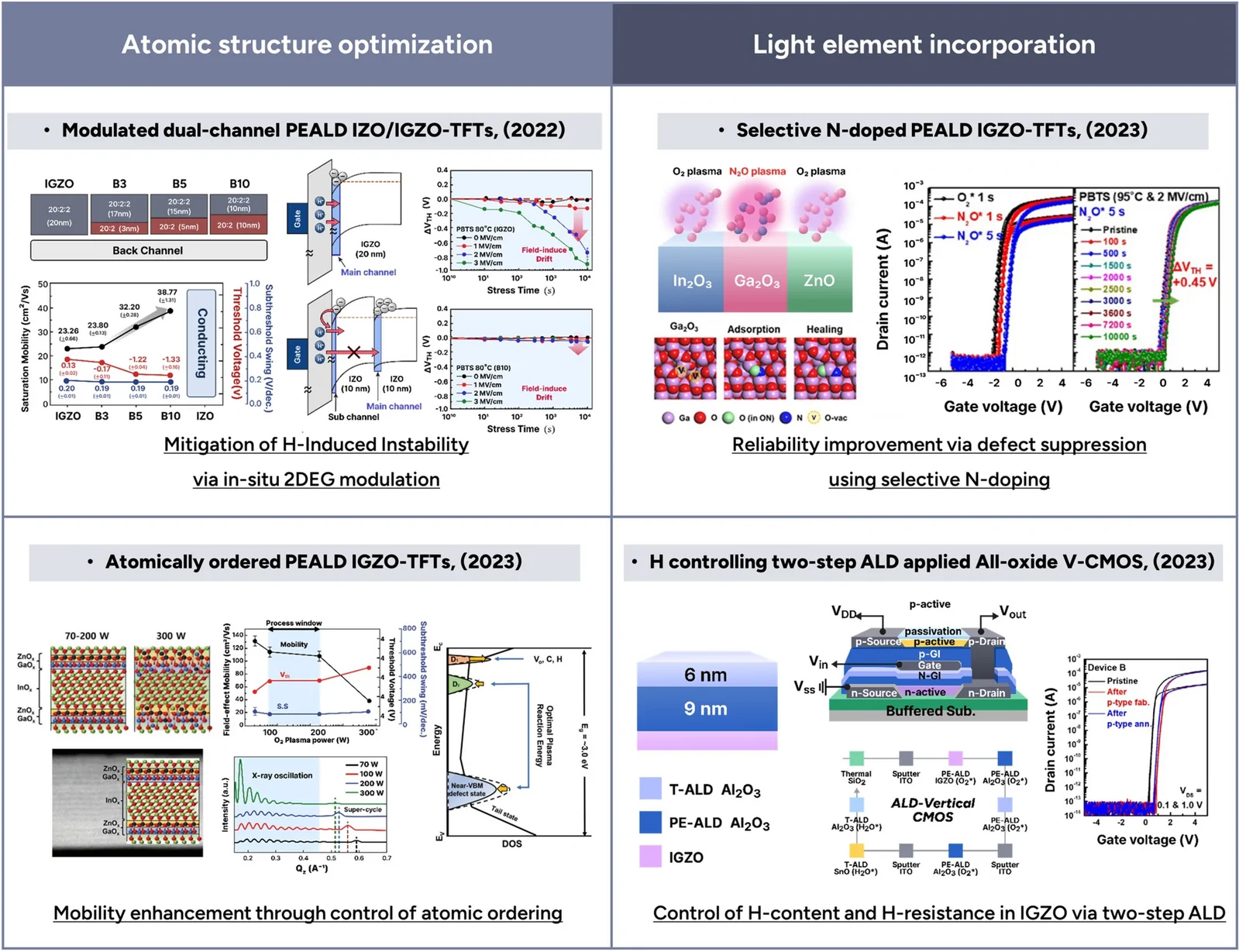
Recent strategies for enhancing the mobility and reliability of oxide–semiconductor FETs through atomic structure optimization (left) and light element incorporation. (right). Reproduced with permission [125, 192, 219, 221]. Copyright 2022, 2023, 2024, John Wiley & Sons. Copyright 2023, ACS Publications

In the pursuit of oxide semiconductors that simultaneously exhibit high mobility and long-term reliability, atomic structure optimization has emerged as an effective strategy. By engineering heterointerfaces and modulating the spatial distribution of constituent layers at the atomic scale, this approach facilitates the formation of spatially confined conduction pathways—such as two-dimensional electron gas (2DEG)-like channels—that decouple charge transport from defect-sensitive or chemically unstable regions. As a result, it enables the mitigation of bias-induced instabilities and the overcoming of the intrinsic mobility–reliability trade-off commonly observed in conventional single-layer architectures [5, 6, 8]. Atomic structure optimization strategies—such as bilayer and nanolaminate architectures—have been uniquely explored using ALD owing to its precise interface control and angstrom-level tunability, enabling the simultaneous realization of high mobility and reliability in oxide TFTs, as illustrated in the left part of Fig. 7.

Kim et al. [219] demonstrated that the optimization of bilayer structures using ALD can alleviate hydrogen-induced instability through in situ modulation of the 2DEG. Researchers established plasma-enhanced ALD (PEALD)-deposited dual-channel IZO/IGZO top-gate TFTs, where nanoscale modulation of the backchannel IZO thickness enabled simultaneous enhancement of mobility and stability. As the IZO layer thickness increased, the main conduction path transitioned from IGZO to high-mobility IZO, achieving a peak mobility of ~ 40 cm^2^ V^−1^ s^−1^, while maintaining a low SS and suppressed threshold voltage shift (Δ*V*_th_ = –0.07 V under PBTS for 10,800 s). Technology computer-aided design simulations confirmed dual-channel formation and revealed a 2DEG-like current path in the backchannel IZO, physically isolated from gate-insulator-induced instability. The hydrogen-resilient IZO layer effectively shielded the active channel from hydrogen diffusion originating in the PEALD-SiO_2_ gate insulator (GI), addressing the abnormal negative *V*_th_ shifts typically observed under bias stress. These results establish ALD-based IZO/IGZO bilayers as a compelling architecture to overcome the conventional mobility–reliability trade-off in oxide TFTs [219].

In terms of laminate structure, Kim et al. [125] developed pseudo-single-crystalline IGZO transistors via PEALD by optimizing the super-cycle sequencing of InO₂ and (Ga,Zn)O layers. By tuning the oxygen plasma power, the researchers achieved ultrahigh field-effect mobility (> 114 cm^2^ V^−1^ s^−1^) and excellent threshold voltage (*V*_th_ ≈ − 0.44 V) and SS characteristics (SS ≈ 90 mV dec^−1^) at the optimal 100 W plasma condition. Excessive plasma power was noted to deteriorate atomic ordering and lead to oxygen over-incorporation, while insufficient power resulted in donor-like defects (H, C), thereby degrading reliability. This study demonstrated the critical role of plasma energy control in achieving defect-minimized, high-mobility OSs suitable for next-generation logic and memory applications [125].

Additionally, as part of atomic structure optimization, ALD-enabled angstrom-scale interlayer and bilayer engineering has been reported as an effective approach to suppress interface defects and form high-quality conduction paths. With respect to atomic interlayer tuning, Li et al. [220] report an AIMD/TCAD-guided sub-nanometer ALD interlayer strategy co-designing the gate-dielectric and contact interfaces. An InO_x_ interlayer, with a thickness of less than 0.5 nm, suppresses interface defects, sustaining SS ≈ 62–64 mV dec^−1^ after 500 °C PDA and PBTS reliability improved to 3.4 mV of |Δ*V*_th_| (1 ks, + 3.5 MV cm^−1^). An IGZO/InO_x_/ITO contact stack exhibits an extracted ≈80 meV barrier consistent with ohmic behavior, and system relevance is demonstrated in 2T0C DRAM (> 10^11^ cycles, *I*_on_/*I*_off_ > 10^8^, retention > 600 s), validating angstrom-level ALD interlayer tuning as a scalable lever to reconcile BEOL thermal budget, reliability, and performance [220]. Collectively, these strategies underscore the potential of ALD-engineered bilayer and laminate structures to precisely balance mobility and reliability by tailoring charge transport pathways and suppressing defect-induced instabilities in OS devices.

#### Light Element Incorporation

In oxide semiconductors, light elements such as hydrogen, carbon, and nitrogen are present due to precursor chemistry and process conditions. The light elements incorporation can be utilized to modulating carrier concentration, passivate subgap states, and stabilize interface environments. Particularly, ALD offers a powerful route to engineer such light-element incorporation at the atomic scale, enabling the optimization of both mobility and reliability in oxide TFTs. Therefore, systematic understanding and deliberate control of light-element chemistry are becoming vital in the development of next-generation semiconductor applications [5, 117, 119]. As shown in the right part of Fig. 7, precise control over the physical and chemical states of light elements has been achieved through the surface reaction of ALD, leading to improved electrical performance and device reliability in oxide TFTs.

Kim et al. [192] introduced a selective nitrogen-doping strategy in PEALD-grown IGZO thin films, resulting in significant improvements in both mobility and reliability of oxide TFTs. By applying N_2_O plasma reactants selectively to individual cation cycles (In, Ga, Zn), the authors demonstrated that nitrogen doping into Ga_2_O_3_ notably suppressed subgap states and improved PBTS reliability, without the mobility degradation observed in In-rich compositions. The optimized device achieved a high field-effect mobility of 106.5 cm^2^ V^−1^ s^−1^ and minimal Δ*V*_th_ shifts (+ 0.45 V PBTS, − 0.10 V NBTS at 95 °C for 10,000 s). Density functional theory calculations and thermal desorption spectroscopy revealed that nitrogen incorporation was governed by the interaction between residual carbon species and ON radicals, enabling controlled passivation of oxygen defects. These findings offer atomic-level insights into composition-specific doping chemistry and provide a viable pathway to overcome the mobility–stability trade-off in amorphous OSs.

As an example of hydrogen doping, Kim et al. [221] introduced a hybrid GI engineered via an in situ two-step ALD process combining PEALD and thermal ALD Al_2_O_3_. This approach enabled high-mobility and hydrogen-resilient IGZO TFTs. By precisely modulating hydrogen, carbon, and oxygen incorporation through the GI stack, the optimized device achieved outstanding electrical performance with *μ*_FE_ = 150.7 cm^2^ V^−1^ s^−1^, SS = 64.0 mV dec^−1^, and Δ*V*_th_ shifts of − 0.43 V (H_2_ annealing) and 0.00 V (PBTS at 95 °C, 10,000 s). The superior reliability was attributable to H-passivation and minimized trap formation owing to tailored chemical bonding states in the hybrid Al_2_O_3_. Furthermore, the device was integrated into an all-oxide vertically stacked CMOS inverter, achieving rail-to-rail operation with a voltage gain of 44.7 V V^−1^ and noise margin of 87.5% at *V*_DD_ = 10 V. These results highlight the potential of in situ hybrid ALD gate engineering for scalable, high-performance oxide-based logic in 3D monolithic integration [221]. Collectively, these findings underscore the critical role of ALD-enabled light element engineering in tailoring defect states and chemical bonding environments, advancing high-mobility and reliable oxide TFTs for next-generation memory and logic electronics.

## Challenges Toward the Use of OSs in Semiconductor Applications

Although the adoption of ALD has enabled continuous improvements in the properties of OSs, several critical challenges remain for their integration into current memory device architectures. The current DRAM industry imposes stringent requirements on the key performance parameters of OS FETs, including *V*_th_, *I*_on_, *I*_off_, SS, and long-term reliability. First, to ensure normally-off operation suitable for cell transistor applications, the devices must operate in enhancement mode with a moderate and well-controlled *V*_th_, typically in the range of approximately 0.3–0.6 V. Enhancement mode operation is favored over depletion mode because it eliminates the need for constant negative gate bias to keep the transistor in the off state, thereby reducing standby power consumption and preventing unnecessary charge leakage from the storage capacitor. Second, a sufficiently high Iₒₙ is required to ensure fast read and write access in DRAM cell operations. *I*_on_ is typically extracted at a gate voltage of *V*_th_ + 1.0 V under the designated drain bias used for cell transistor operation. For current DRAM integration, an *I*_on_ on the order of at least several tens of µA µm^−1^ is generally considered necessary to meet the stringent timing requirements of high-speed sensing and charge transfer, while still maintaining compatibility with low-voltage peripheral circuitry [31, 34]. Third, an ultra-low *I*_off_ is critical for securing long retention times and minimizing standby power consumption in DRAM cell transistors. To suppress charge loss from the storage capacitor and avoid read disturbance in densely integrated arrays, *I*_off_ levels below 1 × 10^–18^ A µm^−1^ are generally required. Such stringent leakage control ensures stable data storage over extended periods, even at scaled gate lengths. Fourth, a steep SS is essential to enable low-voltage operation while maintaining a sufficient on–off current ratio in DRAM cell transistors. In the ideal, defect-free limit governed by Boltzmann carrier statistics, SS approaches 60 mV decade^−1^ at 300 K. Values close to this limit are desirable because they allow the device to switch effectively at reduced gate voltages, thereby lowering dynamic power consumption and easing the voltage requirements for peripheral circuitry. In practical oxide semiconductor FETs, achieving SS near the Boltzmann limit requires minimizing trap densities at the gate dielectric/semiconductor interface and within the bulk channel, which also contributes to enhanced threshold stability under bias stress [222]. Finally, long-term reliability is a critical requirement for DRAM cell transistors, as device characteristics must remain stable over years of continuous operation under elevated temperature and electrical stress. Typical qualification targets demand stability under conditions such as 95 °C and an electric field of approximately 2 MV cm^−1^ for 5–10 years of operation, with minimal degradation in *I*_on_ (within 10%) or *V*_th_ (within Δ*V*_th_ = 30 mV). Reliability poses significant challenges for oxide semiconductor FETs due to two dominant mechanisms: (1) charge trapping in the gate dielectric, which induces *V*_th_ shifts and mobility degradation, and (2) hydrogen diffusion from ALD-grown dielectrics into the semiconductor channel, which becomes increasingly dominant at elevated temperatures. Therefore, ensuring DRAM-compatible reliability requires both dielectric engineering to suppress trap formation and barrier strategies to mitigate high-temperature hydrogen diffusion from the insulator to the channel [223, 224].

**Fig. 8:**
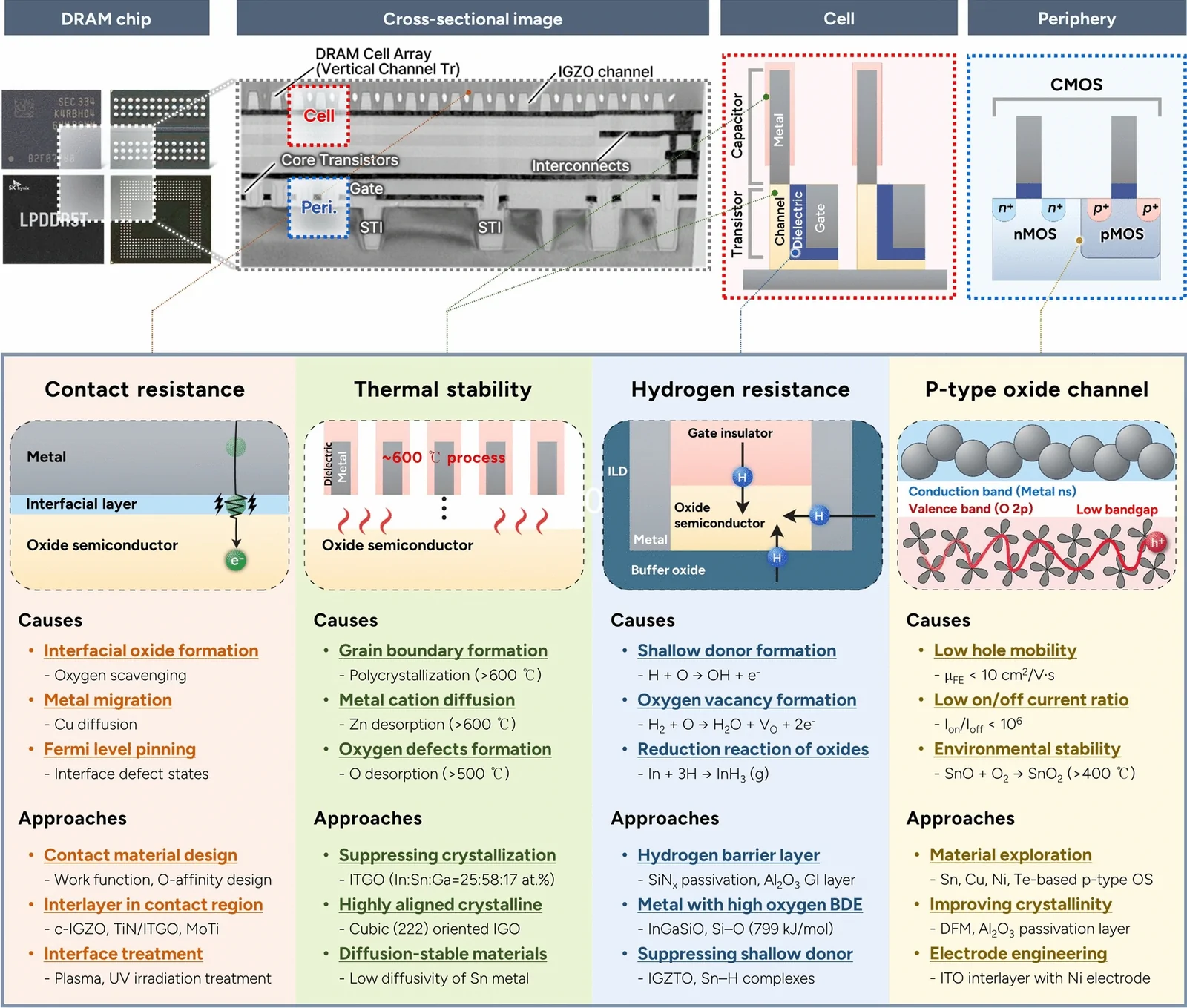
Four key challenges to integrating oxide semiconductors into memory devices: (1) high contact resistance, (2) thermal instability under high-temperature processing, (3) hydrogen-induced electrical degradation, and (4) absence of high-performance p-type oxide semiconductors. It schematically links each challenge to its primary physical/chemical causes and to representative mitigation approaches. The top panels provide device context (chip → cross-section → cell/periphery), while the bottom panels link the causes to the corresponding approaches. DRAM chip images provided by Samsung Electronics and SK Hynix. Reproduced with permission [34]. Copyright 2024, IEEE

In addition to the aforementioned device-level requirements, several critical challenges remain to be addressed. As illustrated in Fig. 8, key issues include high contact resistance, limited thermal stability, poor hydrogen resistance, and insufficient performance of p-type OSs. Extensive research efforts are underway to elucidate the causes of these limitations and to develop effective solutions.

### Contact Resistance

An increase in contact resistance leads to a reduction in cell current, thereby degrading the device’s response speed and power efficiency. In conventional silicon-based memory devices, contact engineering techniques such as silicide control and interlayer insertion have been employed to reduce specific contact resistivity (*ρ*_C_) to below 10^–8^ Ω cm^2^. However, in the application of OS channels to such device architectures, their inherently high *ρ*_C_ (~ 10^–6^ Ω cm^2^) remains a significant bottleneck. In contrast to the display industry, which typically employs structures with larger hole sizes (≥ 10^–8^ cm^2^), the DRAM industry requires contact areas as small as approximately 10^–12^ cm^2^ [225]. This significant reduction in contact size highlights the necessity of developing novel contact technologies specifically tailored to oxide semiconductors. One of the primary factors contributing to contact resistance is the migration of oxygen at the metal–semiconductor interface. For example, IGZO is known to form native oxides at the metal interface, which can modulate the carrier concentration through interfacial oxygen vacancy generation [40, 226, 227]. While moderate oxygen scavenging by the metal enhances electron doping and reduces contact resistance, excessive scavenging may lead to structural degradation, interfacial trap formation, and ultimately increased resistance. In addition, interfacial issues such as metal diffusion and Fermi-level pinning have also been reported to contribute to elevated contact resistance.

One promising approach to addressing these challenges lies in material and structural engineering. According to Lin et al., contact resistance is influenced by several factors, including the work function and oxygen affinity of the metal electrode, as well as the band structure and physical dimensions of the semiconductor channel [227]. Their study highlights the need for a contact structure design based on a comprehensive understanding of these interfacial interactions. A second strategy involves the introduction of an additional interlayer in the contact region. Oxide-based interlayers with low resistivity and high bond dissociation energy can suppress interfacial oxide formation [228]. Subhechha et al. introduced a c-axis aligned crystalline IGZO layer at the IGZO/TiN interface, which served as an effective oxygen-tunneling barrier. By preventing oxidation of the TiN electrode, the contact resistance was effectively reduced. Consequently, the device achieved an *I*_on_ of 24 µA µm^−1^, approaching the industry-required benchmark of over 30 µA µm^−1^.

Additionally, selecting interlayer materials that support band alignment engineering to lower the Schottky barrier and inhibit metal diffusion has proven effective in reducing contact resistance [229–232]. Jeong et al. demonstrated that inserting a TiN/ITGO interlayer between ITO and In_2_O_3_ effectively modulates the contact interface and reduces the Schottky barrier height. By optimizing the interlayer thickness, they achieved a reduction in barrier height from 0.4 to 0.2 eV at an 8 nm ITGO thickness, and significantly improved the *ρ*_C_ from 8.0 × 10^–4^ to 9.0 × 10^–6^ Ω cm^2^ [229]. Kim et al. and Lee et al. reported the use of MoTi and self-assembled monolayer interlayers, respectively, to simultaneously reduce contact resistance and suppress Cu metal diffusion [230, 232].

Another approach involves plasma or chemical treatment of the channel surface [233–236]. Such treatments have been reported to improve contact properties by modulating interface dipoles and passivating subgap states, thereby alleviating Fermi-level pinning. Kim et al. demonstrated that ultraviolet irradiation combined with thermal energy could induce the formation of H_O_⁺ sites coordinated with metal species on the IGZO surface, thereby enabling modulation of the carrier density. As a result, the channel-width-normalized contact resistance between Mo and IGZO, extracted using the transmission line method (TLM), was reduced from 13.0 to 9.4 Ω cm. Similarly, Knobelspies et al. investigated the effect of different plasma gas treatments at the IGZO/Ti–Au interface on contact resistance. They reported that CF_4_-based plasma treatment significantly reduced the contact resistance—by a factor of 24.2—through fluorine-induced passivation of oxygen vacancies, which suppressed trap sites and generated additional free electrons.

Despite these efforts, OSs still exhibit higher contact resistance compared with that of silicon-based systems. Moreover, further enhancement in the thermal stability of contact interfacial layers and conditions is essential to ensure reliability under subsequent high-temperature processing conditions. In addition, considering the 3D architecture of next-generation DRAM, the development of high-performance metal electrode processes that are fully compatible with ALD remains a critical challenge.

### Thermal Stability

In the display industry, for glass-based displays, the thermal budget is typically limited to below 400 °C while flexible displays using polymer substrates impose even stricter constraints, often below 250 °C. In contrast, in DRAM devices, cell transistors are subjected to subsequent capacitor formation processes at temperatures exceeding 600 °C. Therefore, the channel materials must maintain stable electrical properties within this thermal budget. However, IGZO has been reported to suffer from several limitations under such high-temperature conditions, including polycrystallization, elemental diffusion, and defect generation [169, 237–239]. Jeong et al. reported that initially amorphous IGZO undergoes randomly oriented polycrystallization beyond 600 °C, leading to significant degradation in electrical performance, including a reduction in *μ*_FE_ from 40.9 to 5.8 cm^2^ V^−1^ s^−1^ and an increase in SS from 68 to 117 mV decade^−1^ [237]. Ryu et al. attributed the decrease in IGZO conductivity above 600 °C to the desorption of Zn atoms, which exhibits a higher diffusivity (4.36 × 10^–7^ cm^2^ s^−1^) compared to In (0.73 × 10^–7^ cm^2^ s^−1^) and Ga (0.95 × 10^–7^ cm^2^ s^−1^) [169]. This Zn desorption was correlated with a positive *V*_th_ shift from 0.18 to 1.15 V and a decrease in *μ*_FE_ from 6.0 to 0.3 cm^2^ V^−1^ s^−1^. These effects lead to threshold voltage instability, degraded field-effect mobility, and low stress reliability in IGZO-based FET applications.

To address these challenges, extensive research efforts have focused on developing OSs with enhanced phase stability at elevated temperatures. One primary strategy involves increasing the crystallization temperature of amorphous oxides by modifying their cation composition. In particular, multicomponent In_2_O_3_-based oxides incorporating elements such as Ga, Zn, Sn, Al, and W have been shown to effectively suppress crystallization [126, 169, 240–242]. Within these multicomponent systems, reducing the In^3+^ cation concentration below a critical threshold further enhances resistance to crystallization. For instance, Ryu et al. proposed ITGO (In:Sn:Ga = 25:58:17 at%) as an alternative to conventional IGZO [169]. This composition not only suppresses crystallization through the incorporation of Sn but also benefits from the inherently low diffusivity of Sn (1.05 × 10^–7^ cm^2^ s^−1^), thereby maintaining phase integrity and stable electrical performance (*V*_th_: –0.03 V, *μ*_FE_: 7.7 cm^2^ V^−1^ s^−1^) even after annealing above 700 °C. The high bond dissociation energy of Sn–O further contributes to thermal stability in the bonding structure and suppresses the formation of oxygen-related defects.

A second strategy for achieving phase stability involves the intentional alignment of crystalline grains during the early stages of film growth. This approach minimizes structural changes during high-temperature processing while preserving high carrier mobility. According to Ryu et al., InGaO with a highly aligned cubic (222) orientation along the out-of-plane direction (In:Ga = 4:1 at%) exhibited negligible structural degradation up to 700 °C, in contrast to randomly oriented films [126]. This phase stability also led to reduced changes in bandgap energy, film density, and oxygen bonding states, ultimately enabling excellent device performance (*V*_th_: –0.65 V, *μ*_FE_: 128.2 cm^2^ V^−1^ s^−1^) at temperatures exceeding 600 °C, which meets the thermal budget requirements of the DRAM industry.

Nonetheless, amorphous oxides inherently suffer from limited mobility, while crystalline oxides face challenges in achieving single-crystalline growth, leading to grain boundary–related nonuniformities. Consequently, the debate over the optimal phase—amorphous versus crystalline—for thermally stable OSs remains an ongoing topic in materials research.

### Hydrogen Resistance

Hydrogen is widely incorporated across various stages of memory device fabrication, particularly during passivation, metallization, and annealing. In conventional Si-based memory devices, the low reactivity of silicon with hydrogen, combined with hydrogen’s ability to effectively passivate dangling bonds, has been beneficial for improving device performance. However, when oxide OS channels are integrated into hydrogen-rich process architectures, hydrogen incorporation can lead to the formation of various defects within the channel. Hydrogen can exist in H_i_^+^, H_O_^+^, and V_O_H states in the OS matrix, generally acting as a shallow donor, and has also been reported to promote the formation of oxygen vacancies [243–245]. In contrast, several reports highlight the ambivalent effects of hydrogen. When introduced in appropriate amounts, hydrogen can passivate weak bonds, deep states, and oxygen vacancies in OSs, leading to improvements in SS and short-term PBTS [243, 246–249]. However, the high thermal budgets characteristic of DRAM fabrication—frequently above 600 °C—facilitate rapid hydrogen diffusion and accelerate defect generation relative to display manufacturing, indicating a clear need for methods that improve hydrogen stability. The primary concern regarding hydrogen exposure is the electrical degradation of OS properties. In DRAM applications, where a tight sensing margin is critical, a *V*_th_ shift within ± 0.1 V is typically required even after hydrogen exposure. This necessitates strict control over hydrogen-related and hydrogen-induced defects that can alter carrier density. A second concern is the physical degradation driven by hydrogen’s strong reducing nature, which can induce metal precipitation and etching phenomena [250, 251]. At temperatures above 600 °C, hydrogen exposure has been shown, both thermodynamically and experimentally, to reduce In_2_O_3_ and SnO_2_ into volatile hydrides such as InH_3_ and SnH_4_.

One approach to mitigate these issues is the introduction of hydrogen barrier layers. Thin films such as SiN_x_ and Al_2_O_3_ can be deposited either on top of the OS channel or at critical interfaces to effectively block hydrogen permeation [221, 252, 253]. According to Kim et al., Al_2_O_3_ used as a gate insulator exhibits a low H_2_ permeability of less than 10^–4^ Barrer [221]. By controlling the carbon, oxygen, and hydrogen content within the Al_2_O_3_ film, they demonstrated improved hydrogen resistance, achieving a Δ*V*_th_ as low as –0.13 V after H_2_ annealing. Another strategy involves material-level engineering by incorporating elements with high hydrogen resistance. For example, the inclusion of elements with high oxygen bond dissociation energy can help suppress the formation of oxygen vacancies under hydrogen-rich environments. Saito et al. introduced Si, which has a significantly higher bond dissociation energy for Si–O (799 kJ mol^−1^) compared to Zn–O (< 250 kJ mol^−1^), into InGaZnO to form InGaSiO [245]. As a result, InGaSiO films maintained semiconducting properties with enhancement mode even after H_2_ annealing at 380 °C, whereas the conventional InGaZnO films underwent severe degradation and changed to depletion mode under the same conditions. Similarly, Sn has been reported to trap hydrogen by forming Sn–H complexes, which suppress the formation of shallow donor-like hydrogen states and contribute to enhanced device stability under bias and thermal stress [254]. However, further studies on the effects and behavior of hydrogen under high-temperature conditions are still required, considering the thermal budget of DRAM fabrication processes. In addition to ensuring *V*_th_ stability, achieving robust H_2_ resistance with improved *μ*_FE_ and stress reliability continues to be a critical research objective.

### P-type Oxide Channels

In memory devices, the peripheral circuitry performs a range of critical functions, including word line control, sensing amplification, and precharge operations. For advanced 3D DRAM integration, OS-based circuit technology is essential. Although OSs are predominantly n-type, NMOS-only implementations exhibit inherent limitations that become more pronounced with higher integration, resulting in reduced signal amplitude and lower efficiency. Consequently, CMOS is generally adopted in the periphery to ensure stable signaling, sufficient noise margin, and dependable control and sensing, underscoring the need for reliable p-type oxide semiconductors. However, existing p-type OSs suffer from several limitations, including low hole mobility (< 10 cm^2^ V^−1^ s^−1^), high *I*_off_, and poor stability [8]. First, the low mobility of p-type OSs originates from their valence bands, which are primarily composed of localized O 2*p* orbitals as shown in Fig. 3b, severely restricting hole transport. Considering that state-of-the-art n-type OSs have demonstrated mobilities exceeding 100 cm^2^ V^−1^ s^−1^, p-type OSs are required to achieve mobilities above approximately 40 cm^2^ V^−1^ s^−1^ to ensure balanced drive currents in CMOS circuits. Second, the high *I*_off_ in p-type OSs is mainly due to their intrinsically low bandgap (< 2.5 eV) and the correspondingly low Schottky barrier height at the metal contact, which facilitates thermionic carrier injection in the off state. For sufficient sensing accuracy and noise immunity in practical applications, the *I*ₒ_n_/*I*ₒ_ff_ ratio should exceed 10^6^, with *I*ₒ_ff_ levels below ~ 10^–14^ A µm^−1^. Finally, the poor stability of p-type OSs arises from the metastable oxidation state of the cation (e.g., Sn^2+^ in SnO, which can be oxidized to Sn^4+^ in SnO_2_) and the high density of hole trap sites associated with localized O 2*p* orbitals. Stress reliability requirements are comparable to those of n-type OSs, typically demanding that the *V*_th_ shift remain within 0.1 V under gate bias stress of 2 MV cm^−1^ at 95 °C for multi-year.

To address these challenges, new material systems based on Cu, Sn, Ni, and Te are being actively investigated [207, 255–261]. P-type channels such as CuO, SnO, and NiO, in which the valence band maximum (VBM) is primarily derived from O 2*p* orbitals, have demonstrated *μ*_FE_ below 10 cm^2^ V^−1^ s^−1^. In contrast, TeO_x_ materials, in which the VBM is primarily composed of Te 5*p* orbitals, have recently been regarded as promising p-type candidates, as the large spatial extent of the 5*p* orbitals can reduce the hole effective mass and thereby enhance mobility. Based on Liu et al., the electrical performance of amorphous Te–TeO_x_ thin-film transistors was significantly enhanced by selenium alloying [261]. Controlling the oxygen content to promote suboxide formation effectively widened the bandgap, which in turn suppressed the off-state current. As a result, the optimized Se-alloyed Te–TeOₓ devices achieved an average hole mobility of ~ 15 cm^2^ V^−1^ s^−1^, an *I*_on_/*I*_off_ ratio of ~ 10^7^, and exhibited excellent bias-stress stability with minimal subthreshold degradation.

To further enhance mobility, material engineering strategies that improve crystallinity are being actively explored. Kim et al. reported that in ALD-grown SnO, adopting a DFM for the precursor supply promoted lateral grain growth and improved the c-axis alignment of the (001)-oriented tetragonal structure, thereby reducing structural defects and enhancing carrier transport [207]. As a result, the optimized SnO TFT achieved a one-order-of-magnitude increase in the *I*_on_/*I*_off_ ratio (7.38 × 10^6^), a higher *μ*_FE_ of 1.86 cm^2^ V^−1^ s^−1^, and a low SS of 0.12 V decade^−1^. Approaches involving the introduction of passivation layers have also been investigated to simultaneously improve carrier mobility and device stability. Kim et al*.* reported that incorporating an in situ ALD-grown Al_2_O_3_ passivation layer on the SnO channel suppressed the formation of thermodynamically stable SnO_2_ phases and facilitated the growth of highly oriented, large-grain SnO domains, thereby reducing grain boundary density and scattering centers [258]. This crystallinity enhancement improved hole transport, yielding a *μ*_FE_ of 2.53 cm^2^ V^−1^ s^−1^. Moreover, the use of conformal passivation layers is particularly advantageous in suppressing extrinsic degradation mechanisms, such as oxygen diffusion or interfacial redox reactions, which are accelerated under bias-temperature stress. This highlights the importance of interface and surface engineering in advancing the stability of p-type oxide TFTs.

Another strategy to enhance the performance of p-type FETs involves electrode and contact engineering. Choi et al. emphasized the importance of selecting electrode materials with an appropriate work function to minimize the Schottky barrier height at the metal–semiconductor interface, thereby facilitating efficient hole injection [259]. They demonstrated that inserting a 5 nm indium tin oxide (ITO) interlayer between the Ni electrode (work function = 4.60 eV) and the p-type SnO channel (4.56 eV) effectively suppressed interfacial redox reactions that otherwise induce the formation of insulating SnO_2_ phases. This interface modification enhanced the *μ*_FE_ (~ 2.5 cm^2^ V^−1^ s^−1^), while lowering the off-current from 3 × 10^–9^ to 2 × 10^–11^ A by mitigating defect-induced leakage pathways. Beyond simple work-function matching, recent studies also emphasize the role of contact-induced dipoles and interfacial phase stabilization, suggesting that contact engineering should be considered not only from an energy-band alignment perspective but also as a means to suppress parasitic chemical reactions at the interface.

Nevertheless, p-type OSs still demonstrate inferior performance compared to conventional p-type Si channels, underscoring the need for effective strategies to achieve key targets such as a field-effect mobility exceeding 40 cm^2^ V^−1^ s^−1^, high-temperature stability (~ 600 °C), and excellent bias temperature stress reliability. Furthermore, establishing ALD processes for implementing promising Te-based materials into 3D architectures remains an unresolved challenge.

### Other Challenging Issues

#### Cross-Parameter Trade-Offs and Co-optimization

Interdependent mitigation strategies for contact resistance, thermal stability, and hydrogen resistance inevitably introduce trade-offs, necessitating a co-optimization approach for balanced device performance. These interdependencies are evident in practical schemes such as interlayer design with oxygen scavenging metals, hydrogen barrier formation, and surface or plasma treatments. For contact resistance, interlayers and oxygen scavenging metals can narrow the interfacial barrier and reduce injection resistance. Still, they also risk interfacial redox reactions and metal diffusion during high-temperature steps, which subsequently perturb *V*_th_ stability. For thermal stability, the same metallurgical activity can drive premature crystallization and interface roughening, degrading the SS and long-term reliability. For hydrogen resistance, barrier layers suppress donor formation and limit threshold drift, yet they may introduce interfacial dipoles and fixed charge that worsen the SS or reinforce Fermi level pinning.

Accordingly, diagnostics must be explicitly tied to each issue: wafer map TLM and cross-bridge Kelvin resistor (CBKR) structures for continuous tracking of contact resistance; accelerated thermal stress and retention style tests for thermal stability; and bias temperature stress under controlled hydrogen exposure for hydrogen-related drift. Taken together, these links motivate a co-optimization strategy that sets an appropriate thermal budget to minimize diffusion time and preserves the intended band alignment and barrier function through deliberate control of layer placement and thickness, with closed-loop feedback from the corresponding electrical monitors.

#### Device-to-device and Wafer-to-wafer Variability

While ALD offers excellent film-level uniformity, translating this advantage into consistent device-level performance across large wafers and deeply stacked arrays remains a significant challenge. In OS FETs, variability in *V*_th_, *μ*_FE_, SS, contact resistance, and reliability arises from coupled process and materials factors. Process side contributors include precursor dosing and purge efficiency, spatial nonuniformity of temperature, pattern density effects, and step coverage; materials side contributors include local differences in cation ratio, oxygen vacancy fraction, residual hydrogen, nanocrystallinity, and interface state density. Interface-related factors—such as interlayer thickness fluctuations, interfacial dipoles, contact roughness, and Fermi level pinning—further degrade uniformity.

To address these issues, variance decomposition across the channel, gate insulator, and contact can be coupled with wafer maps and on-chip monitors—such as CBKR, TLM, and Van der Pauw structures—to quantify corner-to-center trends and distribution tails. At the same time, advanced process control with in situ sensing, optimized wafer rotation and flow zoning, controlled post-treatments, and interlayer engineering that suppresses interfacial oxidation remains necessary. In parallel, unified statistical specifications that link device-level variability to array-level failure tails are required, including quantitative targets for sigma of *V*_th_, contact resistance spread, and interface state density that guarantee sensing margin at scale. Closing the loop from variability monitors to real-time tool control across 300 mm wafers and 3D stacks remains a critical milestone.

#### Long-Term Reliability Beyond Bias-Temperature Stress

While BTI has been widely studied in OS FETs, other degradation mechanisms—particularly hot carrier injection (HCI), random telegraph noise (RTN), and soft error rate (SER)—are gaining increasing importance as devices are scaled and sensing margins become tighter. In contrast to Si-based channel, the wide bandgap and disordered network of OSs shift the degradation focus toward contact-adjacent regions and within the channel, where strong lateral fields and injection-limited transport promote HCI-induced trap creation that drives *V*_th_ drift, transconductance loss, and SS degradation. The intrinsically low carrier density amplifies the impact of a few active traps, producing RTN and discrete current fluctuations that directly erode the read margin, while ionizing events can inject transient charge into high-resistance nodes with long recovery times, increasing SER susceptibility.

To evaluate and mitigate these reliability concerns, a systematic qualification framework is required. This framework combines contact-focused hot carrier stress at elevated temperatures, time-domain noise measurements across millisecond–second scales and temperature ranges, and accelerated stress testing—including radiation, electrical, and thermal transients—to extract soft-error cross sections under realistic bias conditions. Mitigation strategies operate across multiple levels. At the material and process level, emphasis is placed on gate-insulator stacks with low interface state density, balanced hydrogen management, and interlayer engineering to stabilize injection barriers and reduce local field concentration. At the circuit and operation level, noise-robust sensing, adaptive bias or refresh schemes, and error correction with redundancy are essential to maintain array-level reliability.

## Conclusion and Outlook

Oxide semiconductors (OSs) have rapidly evolved from display backplane materials into promising channel candidates for advanced memory architectures. Their unique electronic band structure—characterized by delocalized metal ns orbitals, wide bandgaps, and ultralow leakage—provides an intrinsic advantage for low-power and 3D integration. Recent demonstrations in BEOL, 1T1C, 2T0C, and ferroelectric FET (FeFET) architectures highlight their potential to extend beyond conventional silicon-based scaling limits.

Atomic layer deposition (ALD) has emerged as a pivotal enabler in this transformation. By virtue of the four key characteristics—excellent 3D uniformity, angstrom-scale controllability, rational cation-distribution design, and inherently high film quality—ALD enables the growth of dense, low-defect thin films with clean interfaces. Recent advances—including cation engineering with Sn and W, controlled crystallization of ultrathin In_2_O_3_, bilayer/nanolaminate channel design, and light-element incorporation—have demonstrated significant progress in mobility enhancement, bias stability, and thermal robustness. These findings confirm that ALD not only provides scalable processing but also offers a versatile platform for defect control and atomic-scale material design.

Despite these advances, several challenges must be overcome before OSs can be widely adopted in DRAM and other memory technologies. Key bottlenecks include (i) high contact resistance at the metal–semiconductor interface, (ii) limited thermal stability under > 600 °C processing relevant to capacitor formation, (iii) hydrogen-induced threshold voltage instabilities during high-temperature fabrication, and (iv) the lack of high-mobility, stable p-type oxide semiconductors for CMOS integration. Addressing these issues requires a combination of interface engineering, dopant and defect control, hydrogen barrier design, and exploration of novel p-type systems such as TeOx alloys.

Specifically, across four application classes—BEOL, 1T1C, 2T0C, and FeFET—OSs are required to meet the application targets. For BEOL, it is desirable that they deliver high mobility with large *I*_on_, ultralow *I*_off_, small SS, and appropriate *V*_th_ within a sub-400 °C thermal budget. In 1T1C, OSs should retain transfer characteristics and reliability after high temperature (≥ 600 °C) capacitor processing. In 2T0C, charge retention benefits from very low I_off_ in the read FET, while fast (few–tens of ns) writes are favored by high *I*_on_ and large *g*_m_; in parallel, device/layout choices are encouraged to suppress unintended parasitic capacitances. For FeFETs, suitability for high-aspect-ratio 3D vertical architectures and excellent ferroelectric/OS-channel interfacial compatibility are paramount, design strategies, such as adopting p-type OSs, are required to address hole scarcity in n-type OS channels. Across all applications, lower contact resistance, improvement hydrogen resistance, and ALD compatibility is advantageous to conformally realize CAA/GAA/VCT 3D geometric FETs for continued scaling.

Looking ahead, OSs are expected to play a strategic role in bridging logic and memory integration, particularly in monolithic 3D system architectures where energy efficiency and scaling are paramount. The demonstrated compatibility of ALD-grown OSs with vertical channel transistors, gate-all-around (GAA) FETs, and ferroelectric memory elements highlights their adaptability across diverse device platforms. Moreover, the integration of AI-driven process modeling and data-driven optimization of ALD chemistry offers a path toward accelerating material discovery and device reliability. Practically, AI/data-driven process optimization proceeds along two primary directions, augmented by large language models (LLMs) as an integrating layer. First, ALD recipe optimization uses closed-loop Bayesian optimization and active learning on high-throughput experiments to meet target film metrics [262–264]. Second is precursor/reactant design, based on DFT-derived machine learning, for precursor/reactant properties optimization or HAR conformality prediction [265–268].

In conclusion, ALD-enabled OSs offer a credible path to next-generation semiconductor industry. From a technology-maturity perspective, the present maturity ranges from proof-of-concept and lab-validated prototypes to early pilot-line (pre-production) demonstration for BEOL/memory-compatible OS channels and selector devices, enabling near-term deployment in low-leakage selectors and peripheral circuits and positioning the technology for mid-term adoption in 1T1C/2T0C/FeFET architectures. Most recently, TSMC Ltd. has substantiated industrial viability by validating yield and reliability at the test-chip level for an ALD-OS–based 1T1C memory array embedded in the BEOL that is fully compatible with advanced logic processes [269]. Additionally, at the 2025 VLSI Symposium, SK hynix positioned oxide semiconductors (OSs) as next-generation channel materials for 4F^2^ memory scaling [270]. However, key risks remain—contact resistance at scaled dimensions, thermal robustness, hydrogen resistance, reliable hole transport, device-to-device and wafer-to-wafer variability, and long-term reliability—yet these are de-riskable through precursor chemistry and ligand design, contact/interface engineering, impurity incorporation and defect management, atomic-scale processing, and system-level integration. With these milestones, the convergence of atomic-scale materials design and device co-optimization is positioned to move OS channels from pilot demonstrations toward qualified, industry-relevant deployment.
